# Key Factors for Improving Predictive Accuracy and Avoiding Overparameterization of the PBPK Absorption Model in Food Effect Studies of Weakly Basic Water-Insoluble Compounds in Immediate Release Formulations

**DOI:** 10.3390/pharmaceutics16101324

**Published:** 2024-10-12

**Authors:** Miao Zhang, Shudong Zhang, Lin Wang, Zhe Zhang, Qin Hu, Dongyang Liu

**Affiliations:** 1 Drug Clinical Trial Center, Peking University Third Hospital, Beijing 100191, China; mia.zhang@northeastern.edu; 2 Department of Pharmaceutical Sciences, School of Pharmacy, Bouve College of Health Sciences, Northeastern University, Boston, MA 02115, USA; 3 NMPA Key Laboratory for Research and Evaluation of Generic Drugs, Beijing Institute for Drug Control, Beijing 102206, China

**Keywords:** PBPK absorption model, food effect, weakly basic water-insoluble compounds, orally administered immediate release formulations, predictive accuracy

## Abstract

**Background/Objectives**: Physiologically based pharmacokinetic (PBPK) absorption models are instrumental for assessing drug absorption prior to clinical food effect studies, though discrepancies in predictive and actual outcomes are observed. This study focused on immediate release formulations of weakly basic water-insoluble compounds, namely rivaroxaban, ticagrelor, and PB-201, to investigate factors that could improve the predictive accuracy of PBPK models regarding food effects. **Methods**: Comprehensive in vitro experimental results provided the basis for the development of mechanistic absorption models, which were then combined with mechanistic disposition models to predict the systemic exposure of the model drugs in both fasted and fed states. **Results**: The developed PBPK models showed moderate to high predictive accuracy for food effects in Caucasian populations. For the Chinese population, the ticagrelor model’s initial overestimation of fed-state absorption was addressed by updating the permeability parameters from Caco-2 cell assays to those derived from parallel artificial membrane permeability assays in FaSSIF and FeSSIF media. This refinement was also applied to the rivaroxaban and ticagrelor models, leading to a more accurate representation of absorption in Caucasians. **Conclusions**: This study highlights the importance of apparent permeability in enhancing the predictive accuracy of PBPK absorption models for weakly basic water-insoluble compounds. Furthermore, the precipitation of PB-201 in the two-stage transfer experiments suggests that precipitation may not be a universal phenomenon for such compounds in vivo. Consequently, the precipitation rate constant, a theoretically essential parameter, should be determined based on experimental evidence to avoid overparameterization and ensure robust predictive accuracy of PBPK models.

## 1. Introduction

Weakly basic water-insoluble compounds frequently face absorption challenges in vivo due to the near-neutral pH of the small intestine, which prompts these compounds to precipitate from their soluble ionic state in the stomach’s acidic environment to a less soluble free form, thus reducing solubility and increasing variability among individuals. Additionally, food consumption further complicates drug absorption by inducing physiological changes in the gastrointestinal (GI) tract, including alterations in gastric emptying, bile secretion, pH balance, and osmolality [1]. Such physiological fluctuations have been implicated in altering the systemic exposure of over 40% of oral pharmaceuticals approved by the US Food and Drug Administration (FDA) or European Medicines Agency (EMA) in the past decade, with a particular emphasis on the bioavailability of weakly basic water-insoluble compounds [2]. Consequently, it becomes imperative to consider the influence of food on drug exposure during the development of new medications and the manufacturing of generics [3,4].

Oral dosage forms theoretically undergo a sequence of processes, including disintegration, dissolution, transfer, and transmembrane passage, before entering systemic circulation [5]. Among existing methodologies, the physiologically based pharmacokinetic (PBPK) absorption model, also known as the physiologically based biopharmaceutical model (PBBM), uniquely integrates these four pivotal processes to predict the in vivo pharmacokinetic behavior. Thus, this model has become a prominent strategy for improving drug formulation and clinical trials by mechanistically predicting bioavailability and food effects, significantly advancing the success rate of drug development [4,6,7,8]. Nonetheless, there are limitations in accurately predicting the absorption characteristics of specific compounds, particularly weakly basic water-insoluble compounds, using the PBPK model approach [9,10]. Approximately 25% of PBPK absorption models fail to quantitatively predict the systemic exposure of active pharmaceutical ingredients (APIs) under a fed state [11,12].

The absorption of these compounds is highly susceptible to fluctuations in GI physiological factors, precipitating consequent changes in solubility, dissolution rate, and the API’s ability to traverse the intestinal mucosal barrier [13]. The unpredictable dynamic changes within the GI tract under both fasted and fed states add complexity to PBPK absorption models when extrapolating absorption characteristics from fasted to fed states. Therefore, improving the predictive performance of PBPK absorption models with low confidence (outside the 2-fold range) in food effect studies remains a focal point for researchers [14,15,16]. To this end, identifying the rate-limiting steps within each process that significantly impact the absorption of a particular API is essential for this advancement. Generally, the precipitation rate constant is considered a crucial parameter for developing a PBPK absorption model with high predictive confidence for weakly basic water-insoluble compounds [11,17]. However, current theories often fail to capture the complexity and variability of the precipitates in the GI environment in vivo. Recent research has indicated that drug permeability assessments conducted in conventional incubation media may be overestimated compared to those performed in biorelevant media [18], potentially leading to the overestimation of the systemic exposure within PBPK models. Hence, it is imperative to comprehensively estimate the crucial parameters affecting API absorption and seamlessly incorporate them into the absorption model to achieve a PBPK model with high predictive performance in food effect studies.

In light of these considerations, this study seeks to develop methods for accurately capturing the absorption characteristics of compounds under both fasted and fed states using PBPK absorption models. To achieve this, we randomly selected three weakly basic water-insoluble compounds with immediate release (IR) dosage forms: rivaroxaban (showing an in vivo food effect), ticagrelor (not exhibiting an in vivo food effect), and PB-201 (demonstrating an in vivo food effect) as model drugs. Subsequently, a series of in vitro experiments were conducted; a robust PBPK model, including both mechanistic absorption and disposition models, was developed; and optimized PBPK models for accurately predicting food effect were built. To summarize, this study encompasses the following key aspects: (i) determining critical in vitro experiment experiments essential for informing mechanistic PBPK absorption model development to avoid model overparameterization and (ii) identifying crucial model parameters for optimizing the PBPK absorption model to realize our investigation goals.

## 2. Materials and Methods

### 2.1. Materials

Rivaroxaban (CAS numbers: 366789-02-8, pKa 13.6 [Drug Bank online], log *p* 1.90 [predicted by ADME predictor, version 10.3, Simulation Plus, Inc., Lancaster, CA, USA], purity ≥ 98%) and ticagrelor (CAS numbers: 274693-27-5, pKa_1_ 2.82 [predicted by ADME predictor], pKa_2_ 0.98 [predicted by ADME predictor], log *p* 3.521 [predicted by ADME predictor], purity ≥ 98%) provided as gifts from SiHuan Pharmaceutical (Beijing, China), along with PB-201 (CAS numbers: Not available during the development stage, pKa 10.41, log *p* 2.102, purity ≥ 98%) [19], which was provided as a gift from PegBio Co., Ltd. (Suzhou, China), were selected as the weakly basic water-insoluble model drugs for this study. Biorelevant powder was purchased from Biorelevant (London, UK). Methanol and acetonitrile were obtained from Merck (Merck KGaA, Darmstadt, Germany). Other chemical reagents used in this manuscript were purchased from Sinopharm Chemical Reagent Co. Ltd. (Shanghai, China). All reagents were of analytical grade and used without further purification. The parallel artificial membrane permeability assay (PAMPA), acceptor sink buffer, and lipid solution were obtained from Pion Inc. (Billerica, MA, USA).

### 2.2. Particle Size Distribution

The particle size of the API directly influences the dissolution rate of formulations [20] and the overall bioavailability of the compound, making it a necessary parameter for establishing the PBPK absorption model. To determine the particle size distributions of the APIs employed in the formulation of rivaroxaban, ticagrelor, and PB-201, the bulk drugs used in the corresponding formulations were measured by the dry method with a Malvern Mastersizer laser diffraction size analyzer (Master 2000, Malvern Instruments Limited, Malvern, UK). Each sample was tested once.

### 2.3. Equilibrium Solubility Studies of APIs in Various Aqueous and Biorelevant Media

To evaluate the effect of pH and bile salt concentration within the physiological range on the solubility of APIs, the equilibrium solubility of APIs in various aqueous and biorelevant media was comprehensively investigated. Specifically, aqueous buffers, including pH 1.0 and pH 2.0 HCl solutions; pH 3.0 and pH 4.5 acetum; and pH 5.0, pH 6.5, and pH 8.0 phosphate buffer solutions (PBS), were prepared according to the guidelines outlined in the Chinese Pharmacopoeia. Additionally, biorelevant media such as FaSSGF, FaSSIF, and FeSSIF were prepared following the prescribed method for biorelevant power [13]. An excess amount of solid drug powder was added to vials containing 20 mL of the corresponding medium, and tests were conducted with continuous stirring at 37 °C for 24 h. Drug concentration was detected every minute through an in situ fiber-optic UV dissolution real-time monitoring system (Pion Scientific Instruments, Billerica, MA, USA). All measurements were performed in triplicate.

### 2.4. Dissolution Tests of Formulations in Biorelevant Media

Dissolution tests employing biorelevant media have gained widespread utilization in formulation development, offering valuable insights into the behavior of APIs and their respective formulations within the GI tract. In this study, dissolution behavior was assessed using the calibrated USP 2 apparatus (Pion Scientific Instruments, Billerica, MA, USA) across various media, including FaSSGF, FaSSIF, and FeSSIF. The fasted gastric environment was simulated with 300 mL of FaSSGF medium [4,21], while FaSSIF and FeSSIF media were used to mimic the intestinal conditions under fasted and fed states, with volumes of 500 and 900 mL, respectively. Dissolution experiments were conducted at a paddle speed of 75 rpm, with the medium temperature maintained at 37 ± 0.5 °C. Sample concentrations were monitored at 1 min intervals via an in situ fiber-optic UV dissolution real-time monitoring system. All experiments were conducted in triplicate.

### 2.5. Two-Stage Biorelevant Dissolution

Because the solubility of weakly basic water-insoluble compounds is higher in the acidic gastric environment compared to the near-neutral intestinal environment, and that bile salts can enhance the solubility of poorly soluble drugs in the intestine, a two-stage dissolution test was performed to explore the drug transfer process from the stomach to the intestine. The transfer apparatus comprised two USP 2 vessels representing the gastric and intestinal chambers, which are interconnected via a peristaltic pump to simulate first-order gastric emptying dynamics. A first-order transfer model, characterized by a half-life of 15 min, was used to replicate average physiological gastric emptying under fasted conditions [22,23,24]. To ensure that the final pH and bile salt concentration in the mixed solution after the transfer of FaSSGF to the intestinal chamber closely matched those in the FaSSIF medium, concentrated FaSSIF and pH 10.2 PBS were simultaneously introduced into the intestinal chamber [25]. Continuous real-time monitoring of sample concentrations was facilitated by the Rainbow DDMS equipped with Pion fiber-optic probes (Pion Scientific Instruments, USA), commencing at the initiation of the transfer process and continuing until 5 min post-transfer. Additional samples in the two-stage dissolution test were analyzed every minute with the same instrument. Furthermore, the paddle speed in each chamber was maintained at 75 rpm, and the temperature was controlled at 37 ± 0.5 °C. The experiment duration was 2 h and was executed three times in parallel.

### 2.6. Apparent Permeability Assay in Biorelevant Media

In light of observed distinctions in the apparent permeability of drugs in biorelevant media compared to standard media [18], the apparent permeability of ticagrelor in FaSSIF and FeSSIF media was measured using the published method with the parallel artificial membrane [13]. The apparent permeability of rivaroxaban and PB-201 (Drug-A) in biorelevant media was referenced from the published results [13].

### 2.7. Development of Mechanistic PBPK Models

In this manuscript, PBPK absorption models were developed using SimCYP software (version 20, Sheffield, UK, Certara Company), while the primary solubility parameters were evaluated utilizing SIVA software (version 4, Sheffield, UK, Certara Company). The comprehensive process of parameter evaluation is as follows: Intrinsic solubility (*S_o_*) and salt limiting solubility factor (SF) values were obtained according to the solubility of the API in different aqueous media and the Henderson–Hasselbalch equation (Equation (1)). Subsequently, the bile micelle to buffer partition coefficients (*K_m_*_:*w*_) in both ionized (*K_m_*_:*w*, *ionized*_) and un-ionized (*K_m_*_:*w*, *un*-*ionized*_) states were evaluated based on the solubility of the APIs in FaSSGF, FaSSIF, and FeSSIF media, employing Equation (1).

(1) $$S_{Tot}=S_{o}\times S_{oscalar}\left(t\right)\times \left(1+\frac{\left[BS\right]\left(t\right)}{C_{H_{2}O}}\times K_{m:w,\;un\text{-}ionized}\right)+S_{i}\left(t\right)\times \left(1+\frac{\left[BS\right]\left(t\right)}{C_{H_{2}O}}\times K_{m:w,\;ionized}\right)+S_{bound,excip}\left(t\right)\;$$

where *t* is the time; *S_Tot_* is the total solubility of the drug in a given medium (aqueous or biorelevant medium); *S_o_* is the intrinsic solubility of the drug; *S_oscalar_* is the scalar for *S_o_* used to capture the increased solubility of the drug in a given medium; *S_i_* is the ionized aqueous solubility; *[BS]* is the bile salt concentration in a given biorelevant medium; *C_H2O_* is the concentration of water; *K_m_*_:*w*, *ionized*/*un*-*ionized*_ are the bile micelle/buffer partition coefficients for ionized and un-ionized species, respectively; and *S_bound_*_, *excip*_ is the amount of drug bound to the excipient at a given excipient concentration [26,27].

Based on the solubility parameters, the crucial dissolution parameters, including the diffusion layer model scalar (*DLM*s), the effective diffusion coefficient (*D_eff_*), and the effective diffusion layer thickness (*h_eff_*), were obtained by fitting the dissolution profiles of the formulation in the FaSSGF, FaSSIF, and FeSSIF media, respectively, according to the dissolution profile equation (Equation (2)).

(2) $$DR\left(t\right)=-N\times {DLM}_{Scalar}\times \frac{D_{eff}\left(t\right)}{h_{eff}\left(t\right)}\times 4\pi a\left(t\right)\times \left(a\left(t\right)+h_{eff}\left(t\right)\right)\times \left(S_{surface}\left(t\right)-C_{bulk}\left(t\right)\right)\;$$

where *t* is the time; *DR* is the dissolution rate; *n* is the number of particles; *DLM_scalar_* is the empirical scalar and the default value was 1; *D_eff_* is the effective diffusion coefficient; *a*(*t*) is the particle radius at time *t*; *h_eff_* (*t*) is the effective diffusion layer thickness at time *t*; *S_surface_* (*t*) is the drug concentration at the particle surface at time *t*; and *C_bulk_* (*t*) is the drug concentration in the bulk solution at time *t* [27].

Precipitation parameters, such as critical supersaturation ratio (CSC) and precipitation rate constant (PRC), were evaluated when precipitation occurs in the neutral intestinal environment after the mixture of solution and undissolved substance is transferred from the gastric chamber in vitro. These parameters were evaluated by fitting the two-stage dissolution profile according to the crystallization kinetics equations [28,29,30]. The absorption module of the PBPK models for rivaroxaban, ticagrelor, and PB-201 was developed using the Advanced Dissolution, Absorption and Metabolism (ADAM) model, which incorporates a mechanistic framework based on drug permeability and the diffusion layer model (DLM). The calibrated permeability of drugs, as determined using the Caco-2 Transwell model, was used to represent the permeability of drugs in vivo [31]. The particle size of the APIs used in the formulation development was incorporated into the Particle Population Balance (PPB) option. The full PBPK model, which includes the predicted steady-state distribution volume (V_ss_) and tissue-plasma partition coefficient (K_p_), was utilized to describe the distribution characteristics of these compounds. The K_p_ scalar was estimated via sensitive analysis to match observed concentration-time (C-T) profiles. The metabolic module for each compound was constructed according to its specific metabolic characteristics. Detailed information on all estimated parameters can be found in Sections S2.1.1, S2.2.1, and S2.3.1 of the Supplementary File.

### 2.8. Validation of PBPK Models

Clinical data from various studies, including single/multiple ascending dose studies (SAD/MAD), drug–drug interaction (DDI) studies, and pharmacokinetic (PK) studies in specific populations, were used for model validation. Since rivaroxaban and ticagrelor have been on the market for several years, clinical data for PBPK model validation were retrieved from the PubMed database (https://pubmed.ncbi.nlm.nih.gov/) using the keywords “rivaroxaban and pharmacokinetics” and “ticagrelor and pharmacokinetics”, respectively. Detailed C-T points from the literature were captured using the WebPlot Digitizer tool (https://apps.automeris.io/wpd4/, accessed on 1 January 2022). The clinical data for PB-201 were identical to the original version [19]. The study designs in the SimCYP simulator mirrored the corresponding real-world clinical trials, including population demographics (age, sex, and ethnicity), dosing regimens, and blood sampling times. Furthermore, all simulations were performed for 10 trials with 10 subjects (total *n* = 100) with the corresponding virtual population. The predictive performance of the PBPK model was estimated with two criteria: (i) the observed C-T profile fell within the 90% confidence interval (CI) of the predicted one, and (ii) the ratios of the main pharmacokinetic parameters (AUC and C_max_) were within a predefined boundary of 0.5–2.0 folds.

### 2.9. Simulation of PK Characteristics in Food Effect Studies

Since the central objective of this manuscript is to accurately capture absorption characteristics using PBPK absorption models in the context of food effect studies involving weakly basic water-insoluble compounds with IR formulations, the validated PBPK models were used to simulate the PK characteristics of drugs in food effect studies. Simulations and predictive performance estimation methods were consistent with those described in Section 2.8.

### 2.10. Optimization of the PBPK Absorption Models

Considering that the multifaceted composition of the PBPK absorption model, consisting of both a permeability module and a formulation module, as well as the observed differences in the apparent permeability of drugs in biorelevant media compared to traditional incubation medium, specific refinements were implemented. In the permeability module, the apparent permeability values obtained from the Caco-2 cell line were replaced with estimated apparent permeability values derived from the parallel artificial membrane in FaSSIF medium to optimize the PBPK absorption model under fasted state. The V_ss_ in the distribution module was still predicted using the method of Rodgers et al. and the K_p_ scalar was fitted according to the C-T profile under fasted state in the food effect study. Compared to the original model (developed in Section 2.7), the parameters of the metabolism module remained unchanged. Combining the adjusted K_p_ scalar in the optimized fasted PBPK model with the permeability of drugs in FeSSIF medium, a fed-state model was developed. Furthermore, the simulation method and validation criteria employed for the optimized PBPK model were the same as those described in Section 2.8. The predictive performance of the optimized PBPK model was evaluated by comparing the closeness between the observed results (PK parameters and C-T profiles) and the predicted results before and after the model optimization.

## 3. Results

### 3.1. Particle Size Distribution

The ratio between particle size and diffusion layer thickness is crucial in determining particle dissolution characteristics [20,32]. Consequently, an estimation of the particle size for the API utilized in formulation development was conducted to serve as the initial value for the PBPK absorption model. The particle size distributions of rivaroxaban, ticagrelor, and PB-201 are illustrated in Figure S1, located in Section S1.1 of the Supplementary File. For rivaroxaban, the measurements of D (0.1), D (0.5), and D (0.9) were determined to be 3.809, 7.955, and 15.532 μm, respectively. Ticagrelor exhibited values of 3.045, 6.926, and 13.605 μm for D (0.1), D (0.5), and D (0.9), respectively. Due to the finely dispersed nature of PB-201 particles, only the average diameter was reported, with a volume average particle diameter of 1.697 μm. In the subsequent development of PBPK absorption model, a log-normal distribution function was employed for rivaroxaban and ticagrelor, whereas a “monodispersed” particle size distribution was used for PB-201.

### 3.2. Equilibrium Solubility of the API in Various Aqueous and Biorelevant Media

The equilibrium solubility of rivaroxaban, ticagrelor, and PB-201 was evaluated in various aqueous and biorelevant media over a 24 h period. The solubility of rivaroxaban remained unaffected by changes in pH and bile salt concentration. This stability can be attributed to its high pKa value of 13.6, which means rivaroxaban remains predominantly non-ionized throughout the gastrointestinal tract (pH 1.6 to 6.5). However, the solubility of ticagrelor and PB-201 was significantly influenced by pH, particularly under acidic conditions (pH 1.0). This behavior is consistent with their nature as weakly basic water-insoluble compounds. Additionally, an increase in bile salt concentration markedly enhanced the solubility of ticagrelor and slightly improved that of PB-201. Detailed results are provided in Table S1, found in Section S1.2 of the Supplementary File.

### 3.3. Dissolution Profiles of Formulations in Biorelevant Media

**Figure 1:**
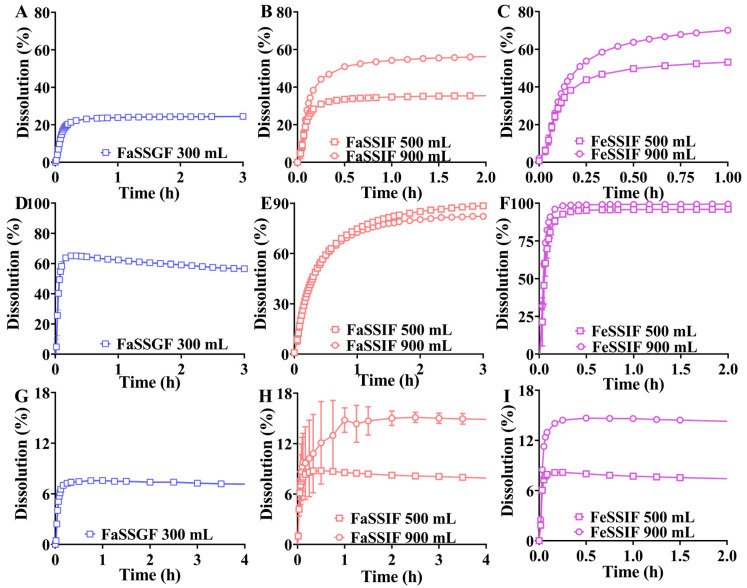
Dissolution profiles of formulations in different dissolution media over time. ((**A**,**D**,**G**) are the dissolution profiles of rivaroxaban, ticagrelor, and PB-201 formulations in 300 mL FaSSGF medium, respectively; (**B**,**E**,**H**) are the dissolution profiles of rivaroxaban, ticagrelor, and PB-201 in 500 and 900 mL FaSSIF medium, respectively; (**C**,**F**,**I**) are the dissolution profiles of rivaroxaban, ticagrelor, and PB-201 in 500 and 900 mL FeSSIF medium, respectively).

The dissolution profiles of rivaroxaban, ticagrelor, and PB-201 in FaSSGF, FaSSIF, and FeSSIF media are depicted in Figure 1. Notably, none of the formulations for these compounds achieved complete dissolution in 300 mL FaSSGF medium (Figure 1A,D,G), indicating that both dissolved and undissolved compounds could be transferred from the stomach to the intestinal lumen in vivo. The dissolution percentages of rivaroxaban and ticagrelor in 500/900 mL FaSSIF and FeSSIF media were higher than in 300 mL FaSSGF medium. Additionally, increased bile salt content improved the dissolution percentages of rivaroxaban and ticagrelor in biorelevant media (Figure 1B,C,E,F). However, a slight difference in the dissolution percentage of ticagrelor between FaSSIF and FeSSIF media was observed (Figure 1E,F), likely due to the high solubility of ticagrelor in both media, as indicated in Table S1. Remarkably, the dissolution percentage of PB-201 remained relatively consistent across 300 mL FaSSGF, 500 mL FaSSIF, and FeSSIF media. Although increasing the dissolution medium volume could increase the dissolution percentage of PB-201 in FaSSIF and FeSSIF media, this effect was comparable between 500 mL (8.24% and 7.42% in FaSSIF and FeSSIF media, respectively) and 900 mL (15.0% and 14.3% in FaSSIF and FeSSIF media, respectively). Therefore, bile salts had minimal influence on the dissolution percentage of PB-201 formulations, consistent with the equilibrium solubility of PB-201 in biorelevant media (Table S1). Given the multiple factors that can affect the dissolution percentage of a given formulation, such as the volume of dissolution medium and bile salts concentration, a comprehensive assessment of in vitro dissolution behavior is essential for understanding its in vivo counterpart.

### 3.4. Transfer Tests of Formulations from the Gastric to the Intestinal Chamber In Vitro

**Figure 2:**
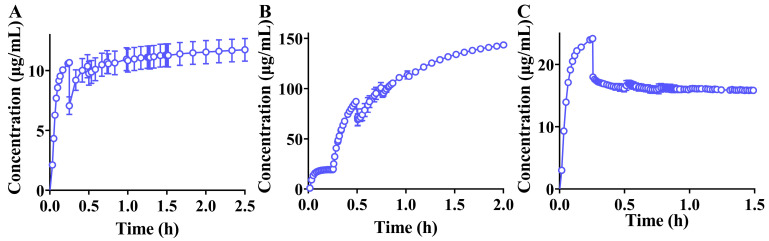
Transfer results of formulations from FaSSGF medium to FaSSIF medium. ((**A**): rivaroxaban formulation; (**B**): ticagrelor formulation; and (**C**): PB-201 formulation).

To simulate the transfer of compounds from the stomach to the intestine, a peristaltic pump interconnected two dissolution vessels, facilitating the in vitro transfer of contents from the gastric to the intestinal chamber. The transfer results are shown in Figure 2. Following the commencement of the transfer (15 min on the abscissa axis), the concentration of rivaroxaban in the FaSSIF medium was lower than that in the FaSSGF medium. Interestingly, the concentration of rivaroxaban in the FaSSIF medium, with a peddle speed of 75 rpm, exhibited a continuous increase post-transfer (Figure 2A). The concentration of ticagrelor in FaSSIF medium demonstrated an upward trend both at the initiation and conclusion of the transfer (Figure 2B), likely due to the high solubility of ticagrelor in FaSSIF medium. Upon transferring PB-201 from FaSSGF to FaSSIF medium, the concentration of PB-201 continued to decrease (Figure 2C), primarily due to the pH alteration of the dissolution medium. The observed precipitation phenomenon of PB-201 in vitro suggests that dissolved PB-201 may undergo a similar precipitation process after transfer from the stomach to the intestine in vivo. Notably, despite rivaroxaban, ticagrelor, and PB-201 all being weakly basic water-insoluble compounds, only PB-201 precipitated after transfer from the gastric to the intestinal chamber (Figure 2C), indicating that not all weakly basic water-insoluble compounds precipitate in vivo. Therefore, evaluating the equilibrium solubility of the drug in various aqueous media at different pH levels and in biorelevant media, coupled with in vitro transfer tests, is crucial for understanding whether the precipitation behavior of weakly basic formulations occurs in vivo.

### 3.5. Apparent Permeability of Compounds in FaSSIF and FeSSIF Media

The apparent permeability of ticagrelor in biorelevant media was determined using the parallel artificial membrane, with results of 9.02 × 10^−5^ cm/s in FaSSIF and 1.43 × 10^−5^ cm/s in FeSSIF media. The trend in the apparent permeability of ticagrelor in biorelevant media aligns with that observed for rivaroxaban and PB-201 [13].

### 3.6. PBPK Models Development and Validation

**Figure 3:**
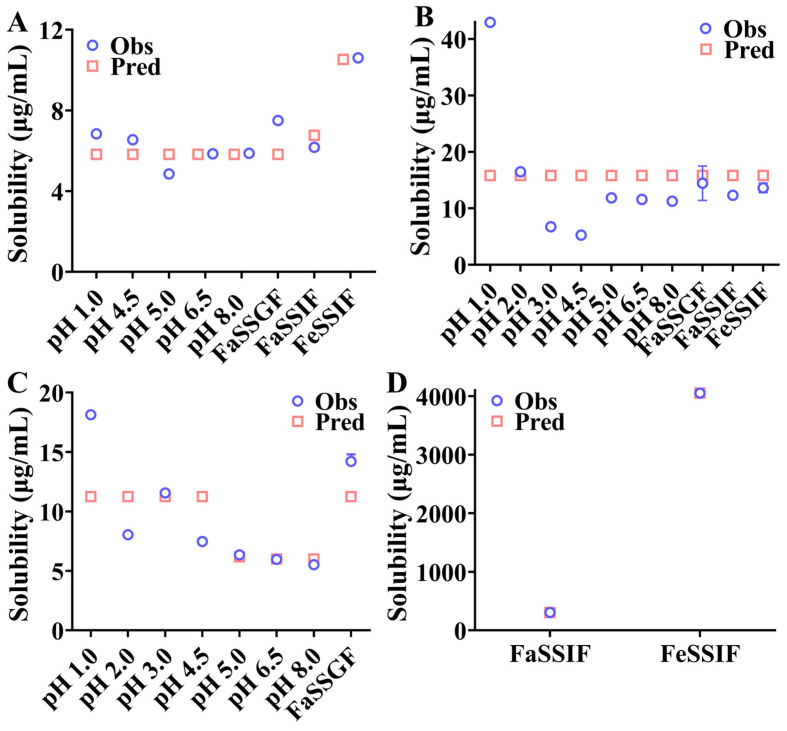
Comparison of predicted and observed solubility of compounds in different media. ((**A**): rivaroxaban; (**B**): PB-201; (**C**,**D**): ticagrelor).

The DLM, coupled with apparent permeability, were used to capture the in vivo absorption characteristics for these three drugs. Parameters including *S_o_*, SF, *K_m_*_:*w*, *ionized*/*un*-*ionized*_*, h_eff_, DLMs*, CSC, and PRC were estimated according to the experimental results and integrated into the DLM. The predicted solubility results in various aqueous and biorelevant media are illustrated in Figure 3. Although the predicted solubility of rivaroxaban and ticagrelor exhibited minor fluctuations around measured values, the overall predicted results were close to experimental results. The effects of bile salt concentration and pH on the solubility of rivaroxaban and ticagrelor were captured. The predicted solubility of PB-201 in aqueous and biorelevant media was generally consistent, with slight deviations from experimental values. However, after excluding PB-201 solubility at pH 1.0 during parameter estimation, the predicted solubility closely matched measured values. This deviation may be attributed to Equation (1)’s inability to simultaneously capture the significant variations in PB-201 solubility at different pH levels, particularly its high solubility in pH 1.0 HCl solution. Based on the solubility parameters, dissolution parameters such as DLM scalar and h_eff_ were evaluated by fitting dissolution profiles of the formulation in FaSSGF, FaSSIF, and FeSSIF media. To enhance the fitting of in vitro dissolution profiles, SF was also estimated during dissolution parameters estimation, likely due to interactions between APIs and excipients affecting APIs solubility in dissolution media. Different dissolution profiles yielded distinct dissolution parameters, and average values of the same parameters were input into the DLM. Moreover, sensitive analyses were performed on these parameters during model development. Figure S2 in Section S1.1 of the Supplementary File illustrates the fitting result of the dissolution profiles in Figure 1. Since only PB-201 precipitated in the transfer test, CSC and PRC of PB-201 were estimated according to experimental results, while CSC and PRC parameters in the PBPK absorption models of rivaroxaban and ticagrelor were set to 1.001 and 0.0001 (the minimum allowable input), respectively. The full PBPK model with the method by Rodgers et al. was used to describe the distribution characteristics of compounds, and the K_p_ scalar was fitted to match the C-T profiles after obtaining metabolism parameters in the metabolic module. The metabolism and elimination parameters of rivaroxaban, ticagrelor, and PB-201 were derived from in vitro experimental results and in vivo clinical data, with detailed results presented in the Supplementary File (Tables S2, S4 and S6). Simultaneously, detailed PBPK model validation results are shown in Sections S2.1.2, S2.2.2, and S2.3.2 of the Supplementary File.

### 3.7. Simulation and Validation Results of Rivaroxaban, Ticagrelor, and PB-201 in Food Effect Studies

#### 3.7.1. Simulation and Validation of Rivaroxaban PBPK Model

**Figure 4:**
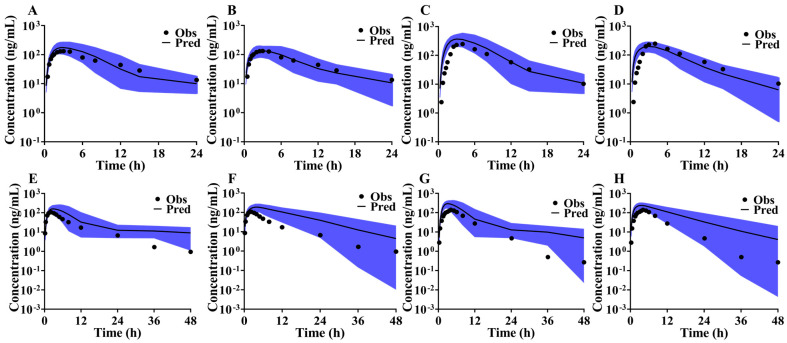
Validation results of the rivaroxaban food effect study in Caucasian and Chinese populations ((**A**,**C**): simulation results of the rivaroxaban PBPK model under fasted and fed states in Caucasians; (**B**,**D**): simulation results of the optimized rivaroxaban PBPK model under fasted and fed states in Caucasians; (**E**,**G**): simulation results of the rivaroxaban PBPK model under fasted and fed states in Chinese; (**F**,**H**): simulation results of the optimized rivaroxaban PBPK model under fasted and fed states in Chinese; the black line is the predicted value, the dark dots are the observed values, and the blue area is the 90% CI of the predicted results).

PK data from food effect studies of rivaroxaban in Caucasians [33] and the bioequivalence studies in Chinese population [34] were sourced from the PubMed database to validate the PBPK model’s capability in capturing absorption characteristics. The simulation results, depicted in Figure 4, demonstrate consistency between predicted and observed PK profiles for both Caucasians (Figure 4A,C) and Chinese populations (Figure 4E,G) in fasted and fed states. The ratios of predicted to observed AUC and C_max_ were 1.74 and 1.26, respectively, in the food effect study, and 1.93 and 1.67, respectively, in the bioequivalence study. These results indicate that the rivaroxaban PBPK model can simulate systemic exposure under fed conditions with moderate confidence.

#### 3.7.2. Simulation and Validation of Ticagrelor PBPK Model

**Figure 5:**
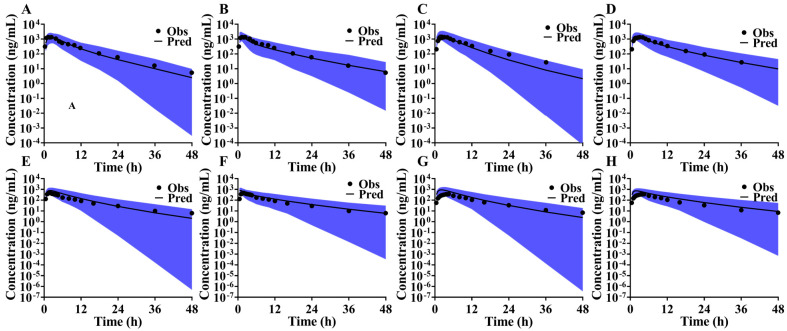
Validation results of the ticagrelor food effect study in Caucasian and Chinese populations ((**A**,**C**): simulation results of the ticagrelor PBPK model under fasted and fed states in Caucasians; (**B**,**D**): simulation results of the optimized ticagrelor PBPK model under fasted and fed states in Caucasians; (**E**,**G**): simulation results of the ticagrelor PBPK model under fasted and fed states in Chinese; (**F**,**H**): simulation results of the optimized ticagrelor PBPK model under fasted and fed states in Chinese; the black line is the predicted value, the dark dots are the observed values, and the blue area is the 90% CI of the predicted results).

Food effect studies of ticagrelor in Caucasians [35] and bioequivalence studies in Chinese populations [36] provided PK data to validating systemic exposure in both a fasted and fed state. The predicted AUC and C_max_ ratios for ticagrelor were 1.01 and 0.83 under fasted conditions, and 1.06 and 1.25 under fed conditions, respectively. For its active metabolite, the AUC and C_max_ ratios were 0.97 and 0.76 under fasted conditions, and 1.17 and 1.19 under fed conditions, respectively. The observed C-T points closely aligned with the predicted PK profiles (Figure 5A,C), affirming high confidence in the ticagrelor PBPK model for predicting systemic exposure under fed conditions in Caucasians. However, in the bioequivalence study, the AUC ratios for ticagrelor in fasted and fed states were 1.73 and 1.99, respectively, with C_max_ ratios being 1.49 and 2.19, respectively. Although the predicted PK profiles were generally consistent with observed results (Figure 5E,G), the ticagrelor PBPK model exhibited limitations in accurately predicting systemic exposure under fed conditions in Chinese subjects.

#### 3.7.3. Simulation and Validation of PB-201 PBPK Model

**Figure 6:**
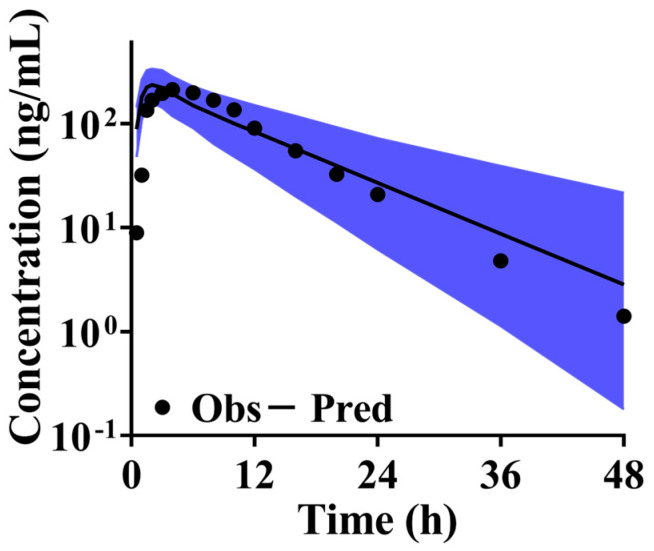
Validation results of PB-201 under fed state in Caucasians (The black line is the predicted value, the dark spots are the observed values, and the blue area is the 90% CI of the predicted results).

The systemic exposure of PB-201 under fed conditions was estimated, with the ratios of predicted to observed AUC and C_max_ being 1.10 and 0.96, respectively. Importantly, the observed C-T points fell within the 90% CI of the predicted profile (Figure 6). Thus, the PB-201 model demonstrates high confidence in capturing the PK characteristics of PB-201 under fed conditions in Caucasians.

### 3.8. Optimization of PBPK Absorption Models

**Figure 7:**
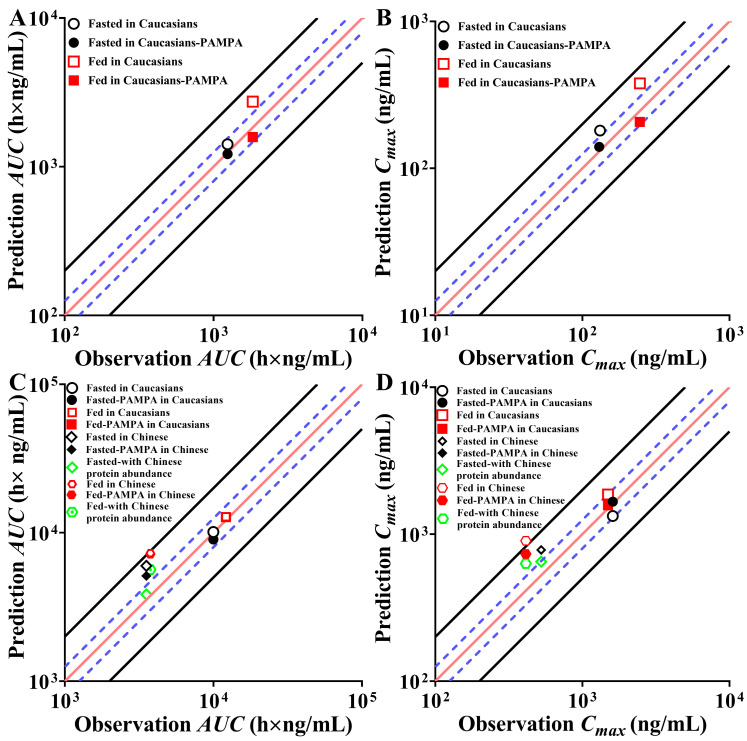
Comparison of PK parameters of rivaroxaban (**A**,**B**) and ticagrelor (**C**,**D**) before and after model optimization. (Note: Fasted/Fed-PAMPA represents the predicted results of the optimized PBPK model, and other predicted results are obtained before the model optimization; black solid lines: predefined 2-fold boundary; blue dashed lines: predefined 1.25-fold boundary; red solid line: indicates the reference line where predicted values align with observed values).

To improve the predictive performance of the rivaroxaban and ticagrelor PBPK models in predicting the effect of food on systemic exposure, the apparent permeability of both drugs in the FaSSIF medium (results in Section 3.5) was incorporated into the models under fasted conditions. The optimized PBPK model parameters for rivaroxaban and ticagrelor are detailed in Tables S3 and S5 of the Supplementary File, respectively. All observed C-T points of rivaroxaban and ticagrelor in “Section 3.7” remained within the 90% CI of the predicted profiles generated by the optimized PBPK model. Comparative results before and after model optimization are shown in Figure 4 and Figure 5. For rivaroxaban in Caucasians, the predicted to observed AUC and C_max_ ratios under fasted and fed states fell within the 0.80- to 1.25-fold range, indicating a significant improvement in the model’s predictive performance regarding food effects in this population. However, the model’s predictive accuracy did not improve for fasted and fed states in Chinese volunteers (Figure 4F,H), even after fitting the K_p_ scalar to the PK profile under fasted conditions in this group. Likewise, the optimized ticagrelor PBPK model demonstrated high confidence in accurately capturing absorption characteristics (AUC and C_max_ ratios within 0.90- to 1.05-fold) for Caucasians under both fasted and fed states. The model’s predictive performance also improved for the Chinese population (Figure 5F,H), with AUC and C_max_ ratios of 1.48 and 1.48 under fasted conditions, and 1.95 and 1.78 under fed conditions, respectively. Therefore, incorporating the apparent permeability of compounds in biorelevant media as an optimization element for the PBPK absorption model effectively enhances the prediction of absorption characteristics under both fasted and fed states. Given that the PB-201 PBPK model already accurately described its absorption characteristics under fed conditions, no further optimization was conducted in this manuscript. Comparisons of PK parameters for rivaroxaban and ticagrelor before and after model optimization are depicted in Figure 7.

## 4. Discussion

Food intake can significantly affect the absorption of orally administered drugs through various mechanisms, leading to four possible changes in exposure compared to in vivo drug administration in the fasted state [2,37]. The PBPK absorption model has become increasingly crucial in prospectively understanding the effects of food on the systemic exposure of compounds. However, a gap remains between the theoretical and practical applications of the PBPK absorption model. In this study, weakly basic water-insoluble compounds, specifically rivaroxaban, ticagrelor, and PB-201, were selected as model drugs. A series of in vitro experiments, including solubility evaluations across various pH levels and biorelevant media, dissolution tests in different dissolution volumes and media, transfer tests from the gastric to the intestinal chamber, and permeability tests in biorelevant media, were conducted. These experiments aimed to identify critical factors in developing the PBPK absorption model, delineate parameters that may cause overparameterization and overestimation, and provide strategies for improving the model’s predictive performance, particularly for weakly basic water-insoluble compounds with IR formulations in food effect studies.

Generally, weakly basic water-insoluble compounds exhibit higher solubility in the stomach than in the intestine, leading to the formation of intestinal precipitates [38]. Increased bile salt concentration can enhance the solubility of insoluble compounds, potentially elevating systemic exposure under fed conditions. However, it was observed that pH and bile salts did not impact the solubility of rivaroxaban, while they significantly improved the solubility of ticagrelor. The solubility of PB-201 was dependent only on pH. Interestingly, precipitates were only observed in the transfer test of the PB-201 formulation, despite its solubility being 14.4 μg/mL in FaSSGF and 12.3 μg/mL in FaSSIF media. Conversely, the solubility gradient of rivaroxaban in FaSSGF (7.50 μg/mL) and FaSSIF (6.17 μg/mL) media did not induce precipitate formation, possibly due to the solubilization of excipients. Factors such as fluid volume, GI membrane permeation, and other physiological aspects may also influence precipitate formation in vivo. Therefore, the relationship between solubility difference in FaSSGF and FaSSIF media and precipitates formation is not straightforward. Despite similar solubility profiles for rivaroxaban and PB-201 in FaSSIF and FeSSIF media, food intake significantly affected their exposure. Conversely, although ticagrelor exhibited substantial solubility differences between FaSSIF and FeSSIF media, food intake did not impact its systemic exposure, likely due to its high intestinal solubility. This suggest that dissolution may not be the limiting step for ticagrelor absorption in vivo. Thus, while solubility in biorelevant media can provide some indication of food effects in vivo, it cannot be used as a direct qualitative basis for assessing the impact of food on drug exposure.

The dissolution percentages of rivaroxaban and PB-201 in 500 and 900 mL FaSSIF or FeSSIF media were below 80%, despite meeting new drug application dissolution criteria [39]. In contrast, the dissolution percentages in FaSSGF medium with physiological volume and pH was limited, indicating that recommended dissolution conditions [39] or phosphate buffer solutions with surfactants may not accurately reflect in vivo dissolution behavior. Integrating in vitro solubility and dissolution experimental data into a mechanistic model is crucial for in vitro-in vivo extrapolation, enhancing the understanding of in vitro results and accelerating new drug development. In SimCYP, dissolution profiles in biorelevant media can be directly entered into the model to estimate in vivo dissolution rates. However, this approach is valid only if the dissolution rate remains consistent across different pH levels, dissolution volumes, dissolution media, and other conditions [25,27]. Consequently, it fails to capture the effects of external and internal factors on the in vivo absorption. This study observed that dissolution profiles of the formulations were highly sensitive to dissolution conditions. Therefore, the DLM, which accounts for changes in GI physiological factors and explores the influence of other factors (such as food or disease status) on absorption, was used to capture the in vivo absorption characteristics of rivaroxaban, ticagrelor, and PB-201 [26,27].

The DLM was developed based on the amended Wang and Flanagan equation (Equation (S2) in the Supplementary File, dissolution rate equation) [27], primarily a function of particle size and diffusion layer thickness [20]. Typically, the particle size distribution of many materials post-grinding approximates a lognormal distribution. The smallest particles dissolve rapidly, while larger particles contribute less to the dissolution process. Therefore, the dissolution rate calculated for a single particle size differs from that for the entire particle size range [40]. The lognormal distribution function (polydispersed in DLM) should be used instead of the single particle size function (monodispersed in DLM) to characterize the particle size of APIs in SimCYP, as it more accurately reflects the actual dissolution process. Consequently, the particle size distribution of the API within the formulation must be obtained and employed as a model parameter. Although only the mean radius of PB-201 is available, it is advisable to input it directly into the model as the initial value rather than using the default value or obtaining the value through sensitive analysis [26] because the sensitivity of absorption to particle size decreases with increasing dose or solubility [41]. The larger bar for PB-201 in the FaSSIF medium with 500 mL may be attributed to experimental error, such as the position of the sample in the dissolution vessel. We compared the estimated parameters and found minimal variation across different media and volumes. Given the consistent parameter estimates and the robust predictive performance of the PBPK model in both fasted and fed states (as shown in Figure 6), the high error bar in Figure 1H is unlikely to significantly influence the model outcomes. Moreover, the DLM incorporates a precipitation kinetics model to characterize the precipitation behavior of compounds in vivo. Since rivaroxaban and ticagrelor continued to dissolve after the transfer from FaSSGF to FaSSIF, the CSC and PRC in the SimCYP software were set to 1.001 and 0.0001, respectively, replacing the default values to avoid excessive model parameterization [42]. In practice, the effects of food on the systemic exposure of rivaroxaban and ticagrelor in Caucasians were directly captured by absorption models. These cases suggest that the precipitation rate constant may not always be a pivotal parameter affecting the predictive performance of insoluble drugs, challenging conclusions proposed by others [11]. Combined with the in vitro transfer test results of rivaroxaban, ticagrelor, and PB-201, these findings underscore the significance of transfer tests in understanding the precipitation behavior of weakly basic water-insoluble compounds in vivo. In vitro experiments encompassing particle size, solubility, dissolution, and transfer tests not only deepen our understanding of in vivo formulation behavior but also establish a foundation for obtaining parameters in the absorption model.

To ensure the rationality of the absorption model and avoid overestimation or underestimation, a mechanistic elimination model was also developed and fully validated for each model drug. The ADME characteristics of each compound in DDI studies and/or PK studies in specific populations were simultaneously captured by the corresponding PBPK model, attesting to the robustness of both absorption and disposition models. The PBPK models exhibited moderate to high confidence in predicting the systemic exposure of rivaroxaban, ticagrelor, and PB-201 in healthy Caucasian subjects under fed conditions. The predicted PK parameters of rivaroxaban and ticagrelor were within a 2-fold range of the observed values in healthy Chinese subjects, except for the overestimation of ticagrelor’s C_max_ under fed conditions. Although the PBPK models in this manuscript effectively evaluate the effects of food on systemic exposure in vivo, there remains room for improvement in the predictive performance of the PBPK model in food effects studies. The ADAM model in SimCYP, comprising permeability and formulation modules, utilizes the DLM to characterize formulation behavior in vivo. To improve the predictive performance of the PBPK model for weakly basic water-insoluble compounds with IR formulations in food effect study, optimizing the permeability of compounds in the model is essential, as the DLM is based on scientific and reasonable experimental results. Although permeability is an inherent property of compounds, the apparent permeability of a specific compound is affected by the type of incubation medium in the donor chamber [43]. Considering that compounds in FaSSIF medium generally exhibit higher permeability than those in FeSSIF medium [13,18,44], and the AUC and C_max_ ratios of rivaroxaban and ticagrelor in the fed state surpass those in the fasted state, it is advisable to improve the predictive performance of the PBPK model in food effect studies by using the permeability of the compounds in FaSSIF and FeSSIF media to simulate the systemic exposure of the compounds under a fasted and fed state, respectively. Due to challenges in culturing Caco-2 cell lines with biorelevant media [45], the apparent permeability of rivaroxaban and ticagrelor in both FaSSIF and FeSSIF media were obtained using the PAMPA method, which can yield comparable results to those derived from Caco-2 cell lines [46]. Moreover, the apparent permeability by PAMPA can be linked to the effective permeability in humans using the SimCYP simulator [31]. In this manuscript, the optimized PBPK models demonstrate high confidence in accurately capturing the absorption characteristics of rivaroxaban and ticagrelor under both fasted and fed states in Caucasians and significantly improve the predictive performance of the ticagrelor PBPK model in predicting the PK characteristics under fasted and fed states in Chinese subjects. Accordingly, the apparent permeability, which closely approximates the real physiological state, emerges as a crucial factor for the PBPK absorption model to accurately depict absorption characteristics.

The rivaroxaban and ticagrelor PBPK models, including their optimized versions, consistently overestimated absorption characteristics in the Chinese virtual population, particularly in the fed state. This discrepancy may not stem from the experimental data used in model development. The accuracy of PBPK model predictions relies not only on the authenticity of the model structure and the reliability of experimental data but also hinges on the quality of anatomical, physiological, biological, and genetic data. Currently, there is a lack of literature on GI physiology specific to Chinese subjects. In the absence of Chinese-specific data parameters, Japanese data are primarily referenced, with North European Caucasian data as a secondary choice [47]. Discrepancies in the absorption and distribution physiological parameters between Chinese and Caucasian virtual populations in SimCYP software arise from the assumption that organ size and blood flow are proportionate to body size [47]. Other parameters such as intestinal transit time, gastric emptying time, pH, and bile salts in the GI tract are considered identical in these two virtual populations. Additionally, a scalar of 0.85 is used in the Chinese virtual population to calibrate the smaller liver volume of Chinese individuals compared to Caucasians [47]. However, a scalar of 0.9 may more accurately describe the liver weight of the Chinese virtual population [48]. Considering that there is no significant difference in weight-normalized renal clearance [47], the overestimated AUC and C_max_ in the Chinese virtual population may be primarily caused by inappropriate GI physiology parameters and underestimated liver metabolism. Differences in distribution [49], renal clearance [50], and liver metabolism [47] between Chinese and Japanese populations cannot be neglected. Therefore, it becomes imperative to collect key physiological parameters affecting drug absorption in the Chinese population in future clinical trials. This will be a crucial step in improving the predictive performance of PBPK model for food effect studies in the Chinese virtual population.

The permeability of compounds in biorelevant media was used as an optimization strategy to improve predictive performance, as realized in this manuscript. Although the PAMPA method quantifies this permeability, it may tend to overestimate the permeability of compounds across the intestinal epithelial membrane. PAMPA excels in characterizing the passive diffusion of compounds across the cell membrane [51], ignoring active transport and metabolic mechanism in multifunctional intestinal epithelial cells [52]. Accordingly, improving permeability assays in a biorelevant manner and establishing in vitro–in vivo correlations is an alternative approach to enhance the predictive performance of the PBPK model in food effect studies. Moreover, a dissolved weakly basic water-insoluble compound may precipitate in vivo, and the reabsorption process of the precipitate may differ from that of the drug substance [13]. Currently, complex reabsorption mechanisms have not been included in the model. Therefore, further attention and efforts are needed to improve the predictive performance of PBPK model in food effect studies. This manuscript summarizes the development and optimization methods of PBPK absorption models that can accurately predict the effect of food on the exposure of weakly basic water-insoluble compounds with IR formulations. These methods aim to improve the predictive performance of the PBPK absorption model in food effect studies of such compounds during new drug development processes.

## 5. Conclusions

In this manuscript, three weakly basic water-insoluble compounds with IR dosage forms served as model drugs to explore the development and optimization of PBPK models for predicting the effect of food on systemic exposure. The goal was to achieve high confidence in the predictions, particularly for weakly basic water-insoluble compounds with IR formulations. Based on the thoroughly validated PBPK model and its strong predictive performance regarding PK characteristics under both fasted and fed states, several considerations are crucial for the subsequent development and optimization of PBPK absorption models for such compounds: (i) an in vitro evaluation of precipitates from IR formulations is essential for determining whether to include the precipitation rate constant in the model, and (ii) the apparent permeability of compounds in biorelevant media serves as a key factor for enhancing the predictive performance of PBPK models. Therefore, these case studies (i) highlight that the precipitation process that may not always occur during GI transport for the weakly basic water-insoluble compounds administered as IR formulations; (ii) demonstrate the importance of reasonably excluding the precipitation rate constant for certain compounds and using the permeability of the compound in biorelevant media to avoid over-parameterization and over-estimation of the model, respectively; and (iii) provide new insights for optimizing the PBPK absorption model to improve its predictive performance in food effect studies in the future. Nonetheless, certain limitations, such as the system bias of the PBPK model in predicting the systemic exposure of compounds in both Caucasians and Chinese, and the overestimation of apparent permeability by the PAMPA method, need further refinement. Ongoing efforts should also be directed towards improving the predictive performance of PBPK absorption models in food effect studies.

## Data Availability

The data presented in this study are available on request from the corresponding authors.

## Supplementary Materials

The following supporting information can be downloaded at: https://www.mdpi.com/article/10.3390/pharmaceutics16101324/s1, Figure S1: Particle size distribution of (A) rivaroxaban, (B) ticagrelor and (C) PB-201; Figure S2: The schematic diagram of the fitting result of the dissolution profile; Figure S3: Validation results for rivaroxaban systemic exposure in the absence (A,C,E,G) and presence of CYP3A inhibitors ((B): ketoconazole, (D): clarithromycin, (F): erythrocin and (H): fluconazole); Figure S4: Validation results for systemic exposure of rivaroxaban in the (A) mild hepatic impairment population (B) moderate hepatic impairment population (C) moderate renal impairment population and (D) severe renal impairment population; Figure S5: Validation results for rivaroxaban SAD study in Caucasians; Figure S6: Validation results for the first dose of rivaroxaban in the MAD study in Chinese; Figure S7: Validation results for rivaroxaban in healthy Caucasian geriatrics with different dosages; Figure S8: Validation results for rivaroxaban in healthy Chinese geriatrics with different dosages; Figure S9: Validation results of rivaroxaban in (A) adult males, (B) adult females, (C) elderly males (D) and elderly females; Figure S10: Pharmacokinetic parameters validated results based on PK studies in healthy volunteers, specific populations (A,C), and DDI studies (B,D); Figure S11: Validation results of disposition model for ticagrelor (A) and its activity metabolite (B); Figure S12: The validation results for ticagrelor and its activity metabolite systemic exposure in the absence ((A,B) respectively) and presence of CYP 3A strong inhibitor ketoconazole ((C,D) respectively); Figure S13: The validation results for ticagrelor and its activity metabolite systemic exposure in the absence ((A,B) respectively) and presence of CYP 3A moderate inhibitor diltiazem ((C,D) respectively); Table S14: The validation results for ticagrelor and its activity metabolite systemic exposure in the mild hepatic impairment population ((A,B) respectively) and severe renal impairment population ((C,D) respectively); Figure S15: The validation results for ticagrelor and its activity metabolite SAD study in Caucasians; Figure S16: The validation results for ticagrelor and its activity metabolite PK study in Chinese; Figure S17: Validation results of ticagrelor and its activity metabolite systemic exposure in adult males ((A,B) respectively), adult females ((C,D) respectively), elderly males ((E,F) respectively) and elderly females ((G,H) respectively); Figure S18: Pharmacokinetic parameters validated results of ticagrelor (A,C) and its activity metabolite (B,D); Figure S19: The validated results for PB-201 systemic exposure in the absence (A) and presence (B) of CYP 3A strong inhibitor ketoconazole; Figure S20: The validation results for PB-201 MAD study in Caucasians; Figure S21: The validation results for PB-201 MAD study in Chinese volunteers; Figure S22: Pharmacokinetic parameters validated results of PB-201; Table S1: Equilibrium solubility of rivaroxaban, ticagrelor, and PB-201 in various test media; Table S2: Parameters for rivaroxaban PBPK model development; Table S3: Parameters for optimized rivaroxaban PBPK model; Table S4: Parameters for ticagrelor PBPK model development; Table S5: Parameters for optimized ticagrelor PBPK model; Table S6: Parameters for PB-201 PBPK model development. References [19,53,54,55,56,57,58,59,60,61,62,63,64,65,66,67,68,69,70,71,72,73,74,75,76,77,78] are cited in the Supplementary Materials.
