# Supplementary material for: Key Factors for Improving Predictive Accuracy and Avoiding Overparameterization of the PBPK Absorption Model in Food Effect Studies of Weakly Basic Water-Insoluble Compounds in Immediate Release Formulations
Source: Pharmaceutics. 2024 Oct 12;16(10):1324. doi: 10.3390/pharmaceutics16101324 (PMC11511194; doi:10.3390/pharmaceutics16101324)
Supplement: Supplementary file 1 [file pharmaceutics-16-01324-s001.zip › pharmaceutics-3173007-supplementary.pdf]

S1. Results for model development

S1.1. Figure

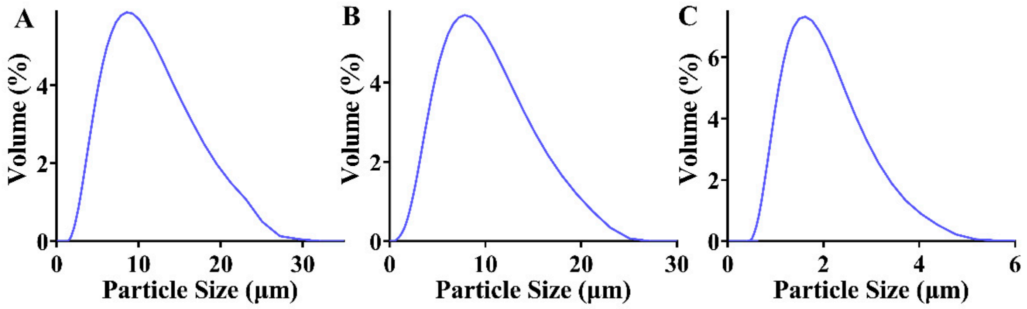

Figure S1. Particle size distribution of (A) rivaroxaban, (B) ticagrelor and (C) PB-201.

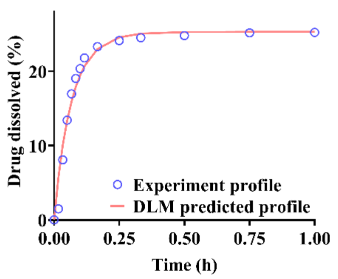

Figure S2. The schematic diagram of the fitting result of the dissolution profile.

S1.2. Table

Table S1. Equilibrium solubility of rivaroxaban, ticagrelor, and PB-201 in various test media.

Model Drug	pH 1.0 HCl (µg/mL)	pH 2.0 HCl (µg/mL)	pH 3.0 Acetic acid buffer (µg/mL)	pH 4.5 Acetic acid buffer (µg/mL)	pH 5.0 PBS (µg/mL)	pH 6.5 PBS (µg/mL)	pH 8.0 PBS (µg/mL)	pH 1.6 FaSSGF (µg/mL)	pH 6.5 FaSSIF (µg/mL)	pH 5.0 FeSSIF (µg/mL)
Rivaroxaban	6.84 (±0.20)	8.18 (±0.03)	10.6 (±0.08)	6.55 (±0.08)	4.85 (±0.10)	5.84 (±0.03)	5.87 (±0.04)	7.50 (±0.28)	6.17 (±0.13)	10.6 (±0.18)
Ticagrelor	18.0 (±0.09)	8.04 (±0.10)	11.6 (±0.14)	7.47 (±0.06)	6.35 (±0.08)	5.97 (±0.05)	5.52 (±0.13)	14.2 (±0.59)	306 (±9.55)	4052 (±49.4)
PB-201	42.9 (±0.50)	16.46 (±0.46)	6.72 (±0.11)	5.24 (±0.06)	11.9 (±0.12)	11.6 (±0.17)	11.2 (±0.16)	14.4 (±3.06)	12.3 (±0.30)	13.8 (±0.85)

S2. PBPK model development and validation

physicochemical parameters for these three model drugs, including protein binding and Blood-to-Plasma (B/P) ratio, were obtained from experimental results, while log P and pKa were estimated based on the molecular formula using Gastro Plus ADMET software (version 10.3, Simulations Plus, US).

S2.1. Rivaroxaban PBPK model development and validation

S2.1.1. Rivaroxaban PBPK model development for metabolism and elimination module

Given that the metabolic fractions of CYP3A4 and CYP2J2 to the systemic clearance of rivaroxaban were approximately 18% and 14% [1], respectively, and liver hydrolases contributed to about 14% of its systemic clearance [2], CL_{int_CYP3A4}, CL_{int_CYP2J2} and CL_{HLM} were fitted according to the metabolic fraction of rivaroxaban *in vivo* and concentration-time profiles from the SAD clinical trial in Caucasian subjects under fasted state [3]. Considering that rivaroxaban was a substrate of P-gp transporter [4], and that about 36% and 7% of the parent drug were directly excreted by the kidney and liver, respectively [1], rivaroxaban may undergo active efflux through the kidney and liver *in vivo* [2]. Consequently, permeability-limited kidney and liver models were employed to account for the active efflux mechanism of P-gp for rivaroxaban. These models include both passive diffusion and active transport. Clearances for passive diffusion in the kidney and liver was calculated using Equation S1 [5] and Equation S2 [6], respectively.

$$CL_{PD,i} = 2 \times P_{app} \times TSA_i \quad (S1)$$

where $CL_{PD,i}$ is the passive diffusion clearance of the drug in compartment *i*, P_{app} is the apparent permeability of the drug, and the TSA_i is the regional tubular surface area of compartment *i* [5].

$$Log\ CL_{PD} = 0.82 \times log\ D_{7.4} + 0.5 \quad (S2)$$

where CL_{PD} is the passive diffusional clearance of APIs in the liver, and *log D 7.4* is the oil-water partition coefficient at pH 7.4 [6]

Furthermore, P-gp efflux clearances in the kidney and liver were fitted based on passive diffusion mechanism and the percentage contribution of parent drug excretion in urine and feces. The detailed PBPK model parameters for rivaroxaban can be found in Table S2.

Table S2. Parameters for rivaroxaban PBPK model development.

Parameter	Input Value	Source
Physicochemical properties		
Molecular weight (g/mol)	435.89	
log *P*	1.90	Predicted
Compound type	Monoprotic base	
pK_a	13.6	Drug Bank online
Blood-to-plasma partition ratio	0.714	Measured [7]
Fraction unbound in plasma	0.065	Measured [2]
Absorption		
Absorption model	ADAM	
Permeability Assay	Caco-2	
Apical pH : Basolateral pH	7.4 : 7.4	
Activity	Passive&Active	
Caco-2(10^{-6}cm/s) (Rivaroxaban)	8.103	Measured [4]

Caco-2(10^{-6}cm/s) (Propranolol)	31	Measured [4]
Caco-2(10^{-6}cm/s) (Cimetidine)	2.36	Measured [4]
DLM Particle Handling Model	Particle Population Balance	
Formulation	Immediate release	
Solid State Specific Parameters	Solid state 1	
Dissolution Type	Solubility	
Solubility Type	Intrinsic solubility	
Solubility (mg/mL)	0.005	Evaluated by SIVA software
S_{o_scalar}	Global	
Global S_{o_scalar}	1.0	Defaulted
Salt Limited Solubility Model	Solubility Factors	
Solubility Factors 1	1.178	Predicted
Supersaturation Precipitation Model	First order	
Precipitation Model	Model 2	
PRC	Global	
PRC (1/h)	1.00E-04	Minimum value
CSR value	1.001	Minimum value
Particle size distribution		
Radius Mean (μm)	20	Predicted
Minimum Radius (μm) (Distribution)	7.940	Predicted
Maximum Radius (μm) (Distribution)	39.907	Predicted
Particle density (g/mL)	1.2	Defaulted
Number of Particle Size Bins (Simulation)	30	Defaulted
Minimum Radius (μm) (Simulation)	0.100	Predicted
Maximum Radius (μm) (Simulation)	43.897	Predicted
DLM Scalar	All segments	
DLM Scalar values	4.575	Evaluated by SIVA software
h_{eff} method selected	Hintz-Johnson	
h_{eff} cut-off value (μm)	40.361	Evaluated by SIVA software
Bile Micelle mediated solubilization	On	
Bile solubilization input type	User	
User log $K_{m:w, neutral}$	3.330	Evaluated by SIVA software
User log $K_{m:w, ion}$	3.894	Evaluated by SIVA software
Distribution		
Distribution model	Full PBPK model	
V_{ss} input type	Predicted	
Prediction Method	Method 2	

Tissue : Plasma Partition Coefficients	Predicted	
K_p scalar	0.03	Fitted
Elimination		
Clearance type	Enzyme kinetics	
Intrinsic clearance of CYP 2J2 (μL/min/pmol)	2.500	Fitted
Intrinsic clearance of CYP 3A4 (μL/min/pmol)	0.027	Fitted
Additional HLM CL_{int} (μL/min/mg protein)	6.000	Fitted
Additional Systemic Clearance (L/h)	0.300	Fitted
Transport		
Organ/Tissue	Kidney	
f_u Kidney Cell Type Urine	0.028	Predicted
f_u Urine	6.31E-07	Fitted
Transporter	ABCB1 (P-gp/MDR1)	
Location	Apical	
Function	Efflux	
$CL_{int,T}$ (μL/min/10^6 cells)	0.001	Fitted
CL_{PD} basal (blood-to-cell) (mL/min/10^6)	0.003	Predicted
PT-S1 Value (mL/min/10^6 cells)	0.003	Predicted
PT-S2 Value (mL/min/10^6 cells)	0.003	Predicted
PT-S3 Value (mL/min/10^6 cells)	0.003	Predicted
Henle's loop Value (mL/min/10^6 cells)	7.74E-05	Predicted
Distal Tubule Value (mL/min/10^6 cells)	9.82E-05	Predicted
Cortical Collect Duct Value (mL/min/10^6 cells)	1.79E-05	Predicted
Medullary Collect Duct Value (mL/min/10^6 cells)	3.64 E-06	Predicted
CL_{PD} apical (blood-to-cell) (mL/min/10^6)	0.003	Predicted
PT-S1 Value (mL/min/10^6 cells)	0.003	Predicted
PT-S2 Value (mL/min/10^6 cells)	0.003	Predicted
PT-S3 Value (mL/min/10^6 cells)	0.003	Predicted
Henle's loop Value (mL/min/10^6 cells)	7.74E-05	Predicted
Distal Tubule Value (mL/min/10^6 cells)	9.82E-05	Predicted
Cortical Collect Duct Value (mL/min/10^6 cells)	1.79E-05	Predicted
Medullary Collect Duct Value (mL/min/10^6 cells)	3.64 E-06	Predicted
Glomerular Filtration Rate	Predicted	
Organ/Tissue	Liver	
Model	Permeability Limited Model	
CL_{PD} (mL/min/10^6)	0.120	Predicted
f_uIW	0.193	Fitted
f_uEW	1	Predicted
Transporter	ABCB1 (P-gp/MDR1)	
Location	Canalicular	

Function	Efflux	
$CL_{int,T}$ (μL/min/million cells)	0.03	Fitted

S2.1.2. Rivaroxaban PBPK model validation

Eight papers, including SAD studies in Caucasian subjects [3], MAD studies in Chinese subjects [8], DDI studies involving rivaroxaban contamination with CYP3A inhibitors [9], PK studies in populations with hepatic impairment [10] and renal impairment [11], and geriatric populations (Caucasian [12] and Chinese [13]) were retrieved from the PubMed database with the keywords of "rivaroxaban and pharmacokinetics" for the validation of the rivaroxaban PBPK model. Given the unavailability of SAD study data in Chinese subjects [8] and the challenge in accurately extracting concentration-time points from the second stage of MAD studies in published literature, the PK data from the first dose in MAD studies were utilized for PBPK model validation, with $AUC_{0\text{-}24h}$ calculated using the trapezoidal area method. The validation results for various scenarios rivaroxaban administration are presented in **Figure S3** to **Figure S9**. In both the absence and presence of CYP3A4 inhibitors, including ketoconazole (**Figure S3A**,**B**), clarithromycin (**Figure S3C**,**D**), erythrocin (**Figure S3E**,**F**), and fluconazole (**Figure S3G**,**H**), the predicted PK profiles of rivaroxaban were matched the observed profiles well, indicating that the rivaroxaban PBPK model could capture the *in vivo* CYP3A4 metabolism mechanism. Moreover, observed concentration-time points of rivaroxaban in populations with hepatic impairment (**Figure S4A**,**B**) and renal impairment (**Figure S4C**,**D**) fell within the 90% CI of the predicted values, and the PK ratios were within 2-fold, suggesting that the permeability-limited liver and kidney models effectively describe the elimination mechanism of rivaroxaban by active transport and passive diffusion *in vivo*. Therefore, the mechanistic elimination pathway of rivaroxaban PBPK model was developed. With the exception of some concentration points in **Figure S5A** (5 mg in Caucasian SAD studies), **Figure S6B** (10 mg in Chinese MAD studies), and **Figure S9D** (PK studies in female geriatric population) deviated from the absorption phase, all observed concentration-time points were within the 90% CI (**Figure S5**, **Figure S6**, **Figure S7**, **Figure S8** and **Figure S9**), indicating that the absorption model effectively capture the absorption characteristics of rivaroxaban. Meanwhile, the PK characteristics of rivaroxaban in healthy volunteers (**Figure S5** and **Figure S6**), geriatrics (**Figure S7** and **Figure S8**), males and females (**Figure S9**) could also be captured by the PBPK model. Furthermore, the PK parameters (AUC and C_{max}) of all the validation results are shown in **Figure S10**, and the PK ratios are predominantly within the predefined boundary. Therefore, the rivaroxaban PBPK model can be used to simulate untested scenarios.

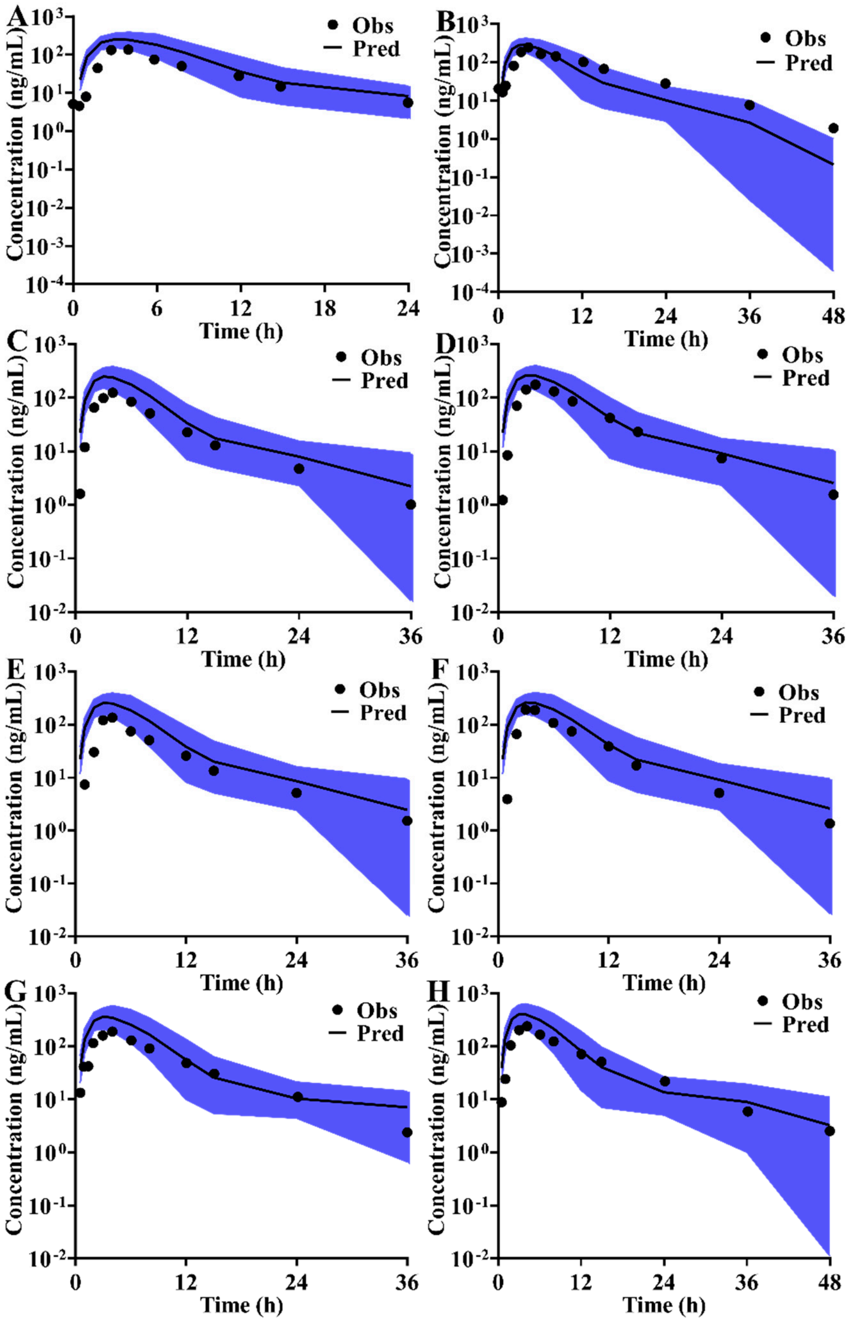

Figure S3. Validation results for rivaroxaban systemic exposure in the absence (A,C,E,G) and presence of CYP3A inhibitors ((B): ketoconazole, (D): clarithromycin, (F): erythrocin and (H): fluconazole) (The black line is the predicted value, the dark spots are the observed values, and the blue range is the 90% CI of the predicted results).

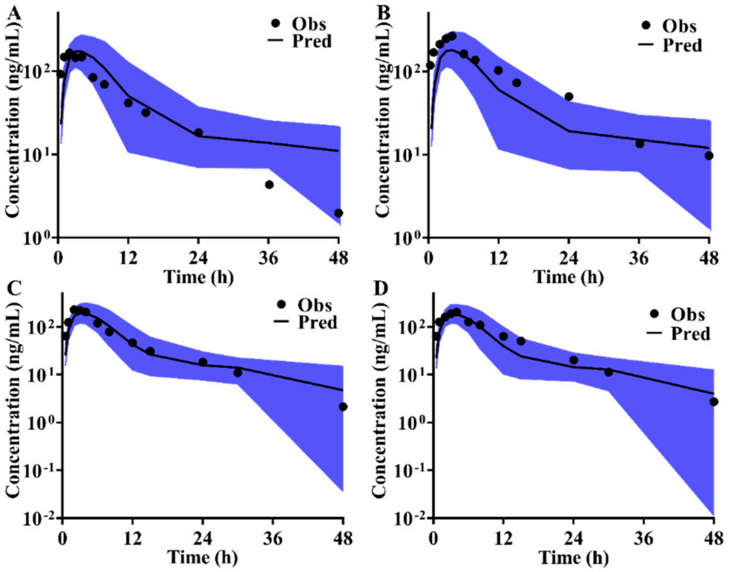

Figure S4. Validation results for systemic exposure of rivaroxaban in the (A) mild hepatic impairment population (B) moderate hepatic impairment population (C) moderate renal impairment population and (D) severe renal impairment population.

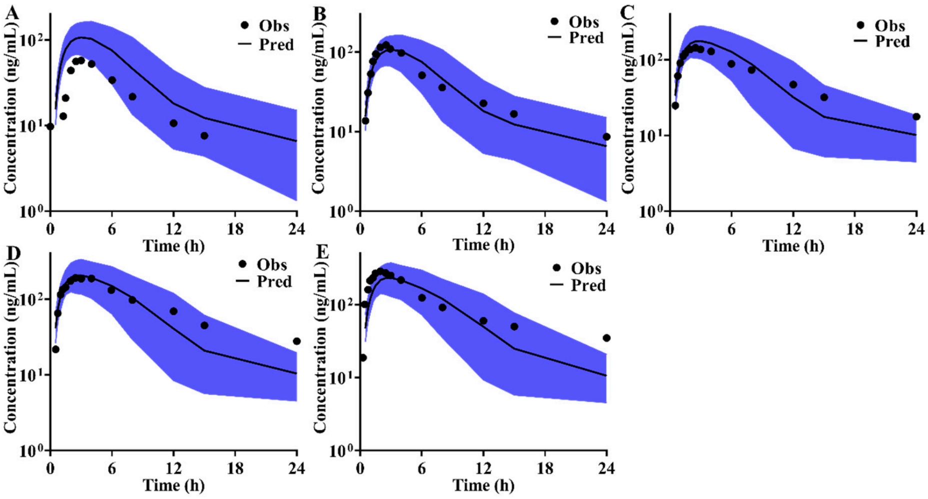

Figure S5. Validation results for rivaroxaban SAD study in Caucasians ((A,B,C,D,E) representing the validation results of rivaroxaban at doses of 5, 10, 20, 40 and 80 mg, respectively).

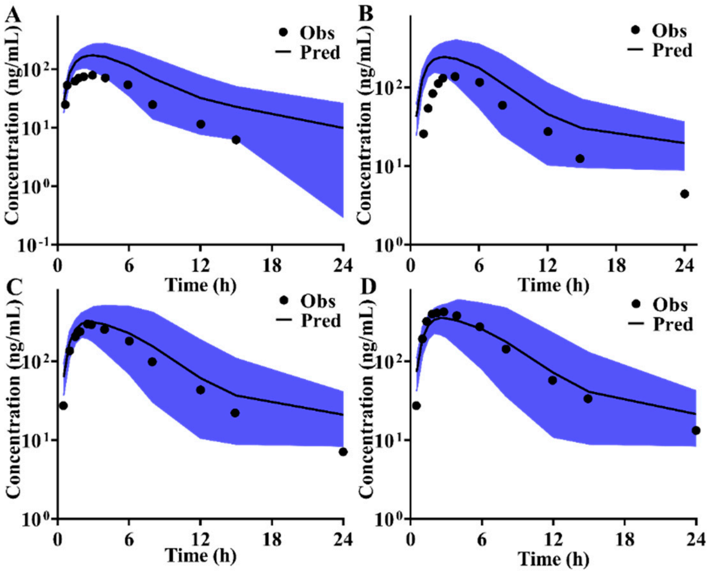

Figure S6. Validation results for the first dose of rivaroxaban in the MAD study in Chinese ((A,B,C,D) representing the validation results of rivaroxaban at doses of 5, 10, 20 and 30 mg, respectively).

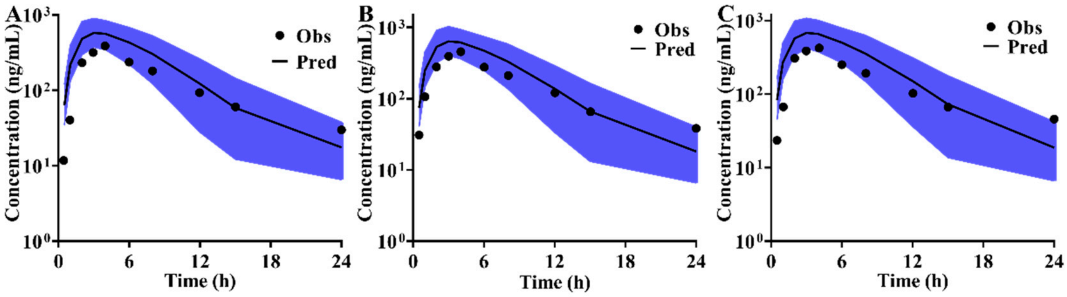

Figure S7. Validation results for rivaroxaban in healthy Caucasian geriatrics with different dosages. ((A): 30 mg, (B): 40 mg and (C): 50 mg).

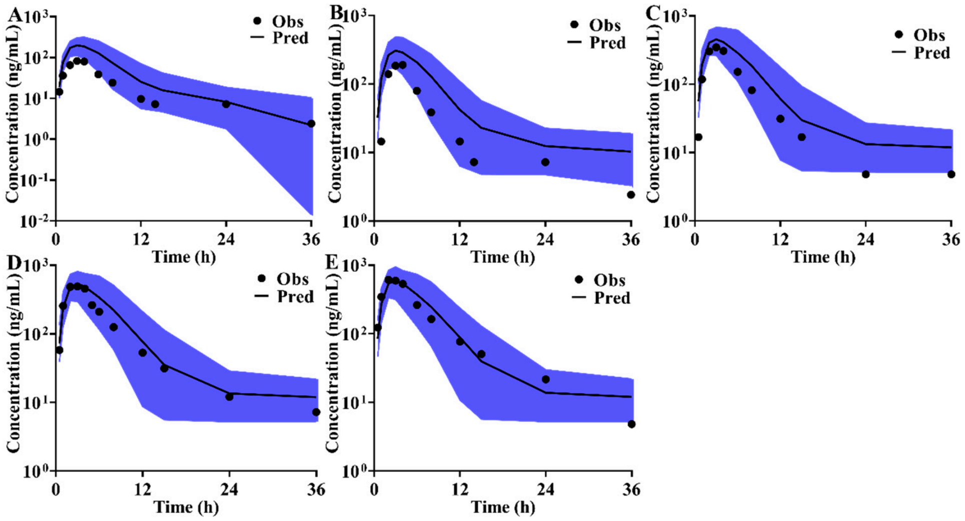

Figure S8. Validation results for rivaroxaban in healthy Chinese geriatrics with different dosages. ((A): 5 mg, (B): 10 mg, (C) :20 mg, (D): 30 mg, and E: 50 mg).

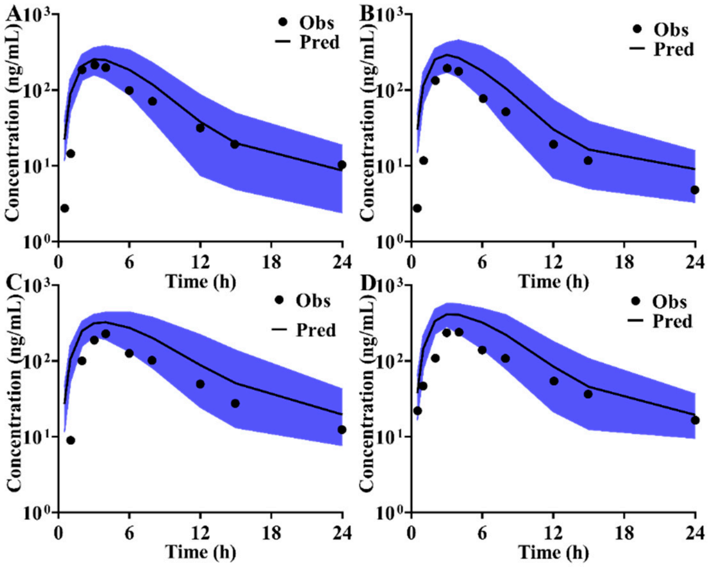

Figure S9. Validation results of rivaroxaban in (A) adult males, (B) adult females, (C) elderly males (D) and elderly females.

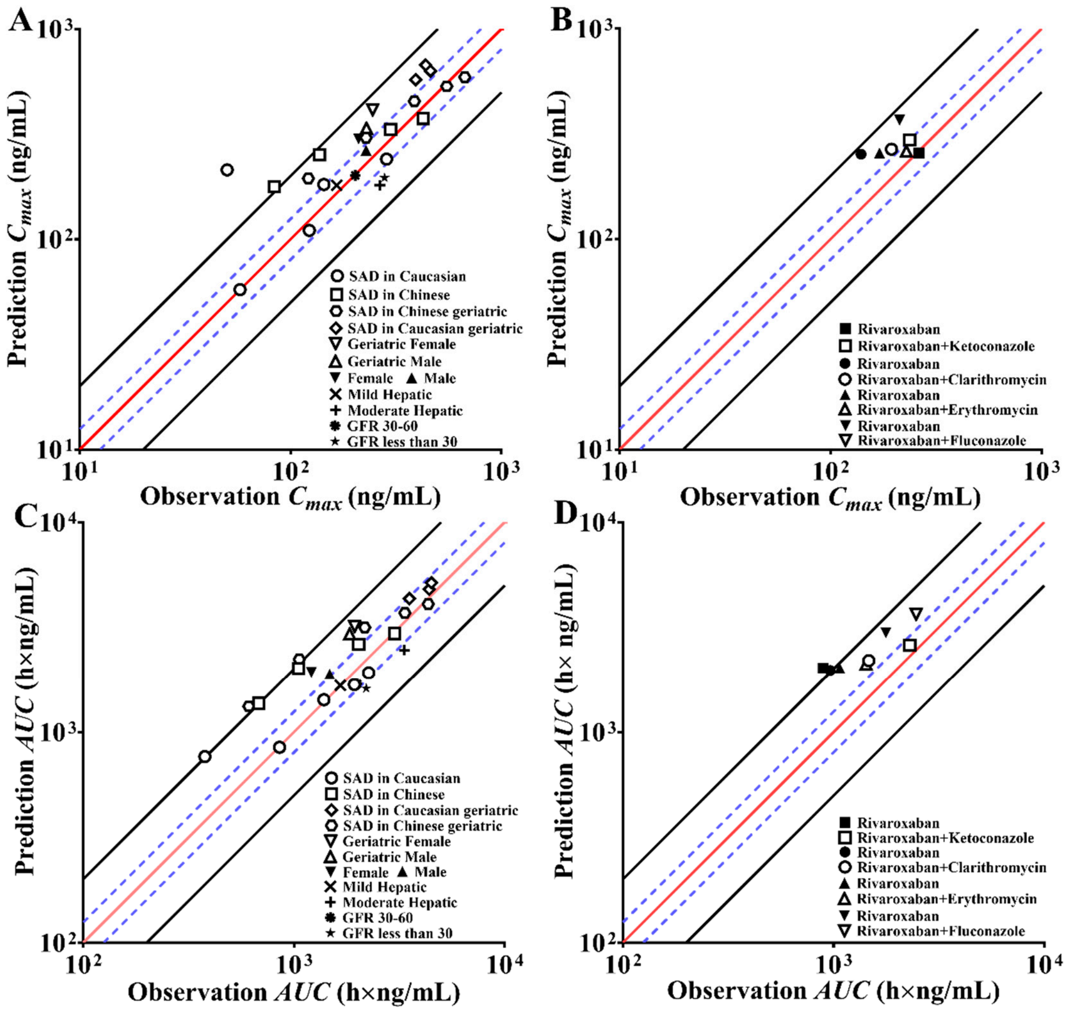

Figure S10. Pharmacokinetic parameters validated results based on PK studies in healthy volunteers, specific populations (A,C), and DDI studies (B,D). (The black solid lines are predefined 2-fold, the blue dashed lines are the predefined 1.25-fold boundary, and the red solid line is axle wire).

S2.1.3. Model parameters for optimized rivaroxaban PBPK model

Table S3. Parameters for optimized rivaroxaban PBPK model.

Parameter	Input Value	Source
Physicochemical properties		
Molecular weight (g/mol)	435.89	
log *P*	1.90	Predicted
Compound type	Monoprotic base	
pK_a	13.6	Drug Bank online
Blood-to-plasma partition ratio	0.714	Measured [7]
Fraction unbound in plasma	0.065	Measured [2]
Absorption		
Absorption model	ADAM	
Permeability Assay	PAMPA	
Apical pH : Basolateral pH	7.4 : 7.4	
PAMPA (10E-06 cm/s)	52.6 (Fasted)/	Measured

	40.80 (Fed)	
DLM Particle Handling Model	Particle Population Balance	
Formulation	Immediate release	
Solid State Specific Parameters	Solid state 1	
Dissolution Type	Solubility	
Solubility Type	Intrinsic solubility	
Solubility (mg/mL)	0.005	Evaluated by SIVA software
S_{o_scalar}	Global	
Global S_{o_scalar}	1.0	Defaulted
Salt Limited Solubility Model	Solubility Factors	
Solubility Factors 1	1.178	Predicted
Supersaturation Precipitation Model	First order	
Precipitation Model	Model 2	
PRC	Global	
PRC (1/h)	1.00E-04	Minimum value
CSR value	1.001	Minimum value
Particle size distribution		
Radius Mean (μm)	20	Predicted
Minimum Radius (μm) (Distribution)	7.940	Predicted
Maximum Radius (μm) (Distribution)	39.907	Predicted
Particle density (g/mL)	1.2	Defaulted
Number of Particle Size Bins (Simulation)	30	Defaulted
Minimum Radius (μm) (Simulation)	0.100	Predicted
Maximum Radius (μm) (Simulation)	43.897	Predicted
DLM Scalar	All segments	
DLM Scalar values	4.575	Evaluated by SIVA software
h_{eff} method selected	Hintz-Johnson	
h_{eff} cut-off value (μm)	40.361	Evaluated by SIVA software
Bile Micelle mediated solubilisation	On	
Bile solubilization input type	User	
User log $K_{m:w,\ neutral}$	3.330	Evaluated by SIVA software
User log $K_{m:w,\ ion}$	3.894	Evaluated by SIVA software
Distribution		
Distribution model	Full PBPK model	
V_{ss} input type	Predicted	
Prediction Method	Method 2	
Tissue : Plasma Partition Coefficients	Predicted	

K_p scalar	0.200	Fitted
Elimination		
Clearance type	Enzyme kinetics	
Intrinsic clearance of CYP 2J2 (μL/min/pmol)	2.500	Fitted
Intrinsic clearance of CYP 3A4 (μL/min/pmol)	0.027	Fitted
Additional HLM CL_{int} (μL/min/mg protein)	6.000	Fitted
Additional Systemic Clearance (L/h)	0.300	Fitted
Transport		
Organ/Tissue	Kidney	
f_u Kidney Cell Type Urine	0.028	Predicted
f_u Urine	6.31E-07	Fitted
Transporter	ABCB1 (P-gp/MDR1)	
Location	Apical	
Function	Efflux	
$CL_{int,T}$ (μL/min/10^6 cells)	0.001	Fitted
CL_{PD} basal (blood-to-cell) (mL/min/10^6)	0.003	Predicted
PT-S1 Value (mL/min/10^6 cells)	0.003	Predicted
PT-S2 Value (mL/min/10^6 cells)	0.003	Predicted
PT-S3 Value (mL/min/10^6 cells)	0.003	Predicted
Henle's loop Value (mL/min/10^6 cells)	7.74E-05	Predicted
Distal Tubule Value (mL/min/10^6 cells)	9.82E-05	Predicted
Cortical Collect Duct Value (mL/min/10^6 cells)	1.79E-05	Predicted
Medullary Collect Duct Value (mL/min/10^6 cells)	3.64 E-06	Predicted
CL_{PD} apical (blood-to-cell) (mL/min/10^6)	0.003	Predicted
PT-S1 Value (mL/min/10^6 cells)	0.003	Predicted
PT-S2 Value (mL/min/10^6 cells)	0.003	Predicted
PT-S3 Value (mL/min/10^6 cells)	0.003	Predicted
Henle's loop Value (mL/min/10^6 cells)	7.74E-05	Predicted
Distal Tubule Value (mL/min/10^6 cells)	9.82E-05	Predicted
Cortical Collect Duct Value (mL/min/10^6 cells)	1.79E-05	Predicted
Medullary Collect Duct Value (mL/min/10^6 cells)	3.64 E-06	Predicted
Glomerular Filtration Rate	Predicted	
Organ/Tissue	Liver	
Model	Permeability Limited Model	
CL_{PD} (mL/min/10^6)	0.120	Predicted
f_uIW	0.193	Fitted
f_uEW	1	Predicted
Transporter	ABCB1 (P-gp/MDR1)	
Location	Canalicular	
Function	Efflux	

$CL_{int,T}$ (μL/min/million cells)	0.03	Fitted

S2.2. Ticagrelor PBPK model development and validation

S2.2.1. Ticagrelor PBPK model development for metabolism and elimination module

Since the active metabolite of ticagrelor (AR-C124910XX) exhibits pharmacological effects similar to ticagrelor *in vivo* [14], the active metabolite model was integrated into the metabolic pathway of ticagrelor. Currently, there is no available CL_{int} information for CYP isoenzymes to describe the *in vitro* metabolic characteristic of ticagrelor. Upon a comprehensive comprehension of ticagrelor's *in vivo* metabolic pathways, the CL_{int_CYP3A4} and CL_{HLM} for both ticagrelor and the active metabolite PBPK models were obtained by fitting PK profiles both before and after ticagrelor co-administration with ketoconazole [15]. Although CYP2D6 might play a role in ticagrelor metabolism, venlafaxine (CYP2D6 inhibitor) showed no significant effect on the systemic exposure of ticagrelor [16]. Therefore, the contributions of other isoenzymes to the clearance of ticagrelor and its active metabolite *in vivo* were characterized using CL_{HLM} in the ticagrelor and active metabolite PBPK model, which was also obtained by fitting PK profiles before and after ticagrelor coadministration with ketoconazole. The CL_R in ticagrelor PBPK model was sourced from the literature [17]. Given that about 0.04% of the active metabolite undergoes renal excretion [17], the process of renal excretion was excluded from the active metabolite model. Refer to **Table S4** for detailed model parameters of ticagrelor and its active metabolite.

Table S4. Parameters for ticagrelor PBPK model development.

Parameter	Input Value	Source
Physicochemical properties		
Molecular weight (g/mol)	522.57	
log *P*	3.521	Predicted
Compound type	Diprotic base	
pK_{a1}	2.82	Predicted
pK_{a2}	0.98	Predicted
Blood-to-plasma partition ratio	0.592	Measured [18]
Fraction unbound in plasma	0.006	Measured [18]
Absorption		
Absorption model	ADAM	
Permeability Assay	Caco-2	
Apical pH : Basolateral pH	7.4 : 7.4	
Activity	Passive&Active	
Caco-2(10^{-6}cm/s) (Ticagrelor)	6.0	Measured [19]
Caco-2(10^{-6}cm/s) (Metoprolol)	15.0	Measured [19]
DLM Particle Handling Model	Particle Population Balance	
Formulation	Immediate release	
Solid State Specific Parameters	Solid state 1	
Dissolution Type	Solubility	
Solubility Type	Intrinsic solubility	
Solubility (mg/mL)	0.006	Evaluated by SIVA software
S_{o_scalar}	Global	

Global S_{o_scalar}	1.0	Defaulted
Salt Limited Solubility Model	Solubility Factors	
Solubility Factors 1	1.875	Evaluated by SIVA software
Supersaturation Precipitation Model	First order	
Precipitation Model	Model 2	
PRC	Global	
PRC (1/h)	1.00E-04	Minimum value
CSR value	1.001	Minimum value
Particle size distribution		
Radius Mean (μm)	3.957	Predicted
Minimum Radius (μm) (Distribution)	0.65	Predicted
Maximum Radius (μm) (Distribution)	13.491	Predicted
Particle density (g/mL)	1.2	Defaulted
Number of Particle Size Bins (Simulation)	30	Defaulted
Minimum Radius (μm) (Simulation)	0.100	Predicted
Maximum Radius (μm) (Simulation)	14.84	Predicted
DLM Scalar	All segments	
DLM Scalar values	0.001	
h_{eff} method selected	Hintz-Johnson	
h_{eff} cut-off value (μm)	2.00E-04	Evaluated by SIVA software
Bile Micelle mediated solubilisation	On	
Bile solubilization input type	User	
User log $K_{m:w,\ neutral}$	5.941	Evaluated by SIVA software
User log $K_{m:w,\ ion}$	8.226	Evaluated by SIVA software
Distribution		
Distribution model	Full PBPK model	
V_{ss} input type	Predicted	
Prediction Method	Method 2	
Tissue : Plasma Partition Coefficients	Predicted	
K_p scalar	0.6	Fitted
Elimination		
Clearance type	Enzyme kinetics	
Intrinsic clearance of CYP 3A4 (μL/min/pmol)	5.00	Fitted
Additional HLM CL_{int} (μL/min/mg protein)	100	Fitted
CL_R typical renal clearance (L/h)	0.00584	Measured [17]
Metabolite		
Physicochemical properties		
Molecular weight (g/mol)	478.5	
log P	3.58	Predicted

Compound type	Diprotic base	
pK_{a1}	2.76	Predicted
pK_{a2}	0.76	Predicted
Blood-to-plasma partition ratio	0.5706	Predicted
Fraction unbound in plasma	0.002	Measured [14]
Distribution		
Distribution model	Full PBPK model	
V_{ss} input type	Predicted	
Prediction Method	Method 2	
Tissue : Plasma Partition Coefficients	Predicted	
K_p scalar	5.5	Fitted
Elimination		
Clearance type	Enzyme kinetics	
Intrinsic clearance of CYP 3A4 (μL/min/pmol)	6.000	Fitted
Additional HLM CL_{int} (μL/min/mg protein)	5000	Fitted

S2.2.2. Ticagrelor PBPK models validation

Clinical data, comprising an absolute bioavailability study [20], SAD studies in healthy Caucasians [21], PK studies in healthy Chinese volunteers [22], DDI studies involving ticagrelor coadministration with ketoconazole [15], diltiazem [15], rifampicin [23], as well as PK studies in populations with hepatic impairment [24], renal impairment [25], and healthy geriatric population [26], were obtained from PubMed database. Since ticagrelor could be directly distributed into the systemic circulation after intravenous (IV) administration, the PK data from the IV study served as the initial validation step for the elimination characteristic of the ticagrelor PBPK model, as depicted in **Figure S11**. The observed values fell within the 90% CI of the predicted PK profiles, and the PK ratios were within the range of 0.5 to 2-fold, indicating that the PBPK models of ticagrelor and its active metabolite could well describe the elimination characteristics. Although the active metabolite PBPK model exhibited limitations in capturing the increased metabolite during IV administration, it successfully aligned with other predicted PK profiles for oral ticagrelor when compared to observations. Thus, further optimization of the metabolite PBPK model was deemed unnecessary.

The parameters related to hepatic metabolism in the ticagrelor PBPK model were obtained by fitting the PK profile in the absence and presence of ketoconazole (**Figure S12**). Subsequently, these parameters were validated by PK data collected from DDI studies involving ticagrelor coadministration with diltiazem (**Figure S13**). The ratios of predicted AUC and C_{max} for ticagrelor and its active metabolite in comparison to observed values were 1.23 and 1.36, 1.28 and 1.12, respectively. Moreover, PK data from ticagrelor co-administered with rifampicin were also used to validate the model. The ratios of predicted AUC and C_{max} for ticagrelor and its active metabolite to observed ones were 0.86 and 0.53, 0.61 and 0.41, respectively. It's noteworthy that ticagrelor serves as a substrate for both CYP3A4 and P-gp, both of which can be induced by rifampicin. Because the P-gp transporter mechanism was not included in the model, the effect of rifampicin on systemic exposure, particularly on the C_{max} of the active metabolite, was underestimated. Despite this limitation, the contribution of CYP3A4 to the systemic clearance was reasonably captured based on other DDI validation results.

The PBPK models of ticagrelor and its active metabolite were also validated by the PK data obtained

from hepatic impairment (**Figure S14A**,**B**) and renal impairment (**Figure S14C**,**D**) studies. The observed values collected from these impairment populations were well around the predicted PK profiles, with the PK ratios (predicted *vs.* observed) of both ticagrelor and its active metabolite falling within the range of 0.80 to 1.25-fold in the hepatic population and within the broader range of 0.75 to 1.56-fold range in the renal impairment population, indicating that the elimination mechanism of ticagrelor was well-matched in the PBPK models. Moreover, the ticagrelor PBPK models had good predictive performance in healthy Caucasian and Chinese volunteers (**Figure S15** and **Figure S16**), adult males (Figure S15 A and B), adult females (**Figure S17C**,**D**), elderly males (**Figure S17E**,**F**), and elderly females (**Figure S17G**,**H**). The PK ratios for these scenarios were almost within the 0.80- to 1.25-fold range (**Figure S18**). Therefore, the mechanistic ticagrelor PBPK models effectively capture the absorption and disposition characteristics, demonstrating its utility for estimating PK characteristics in untested scenarios.

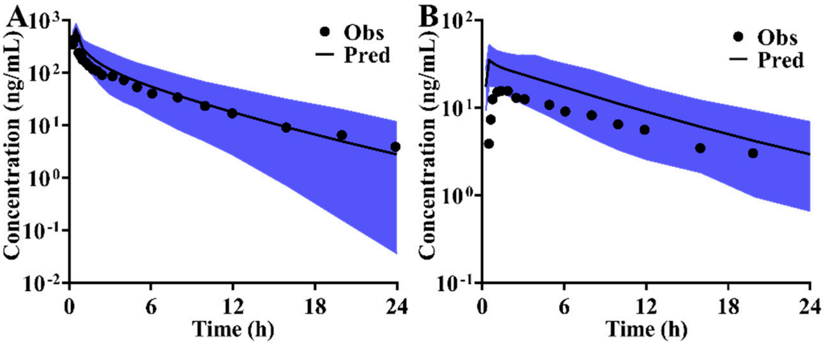

Figure S11. Validation results of disposition model for ticagrelor (A) and its activity metabolite (B).

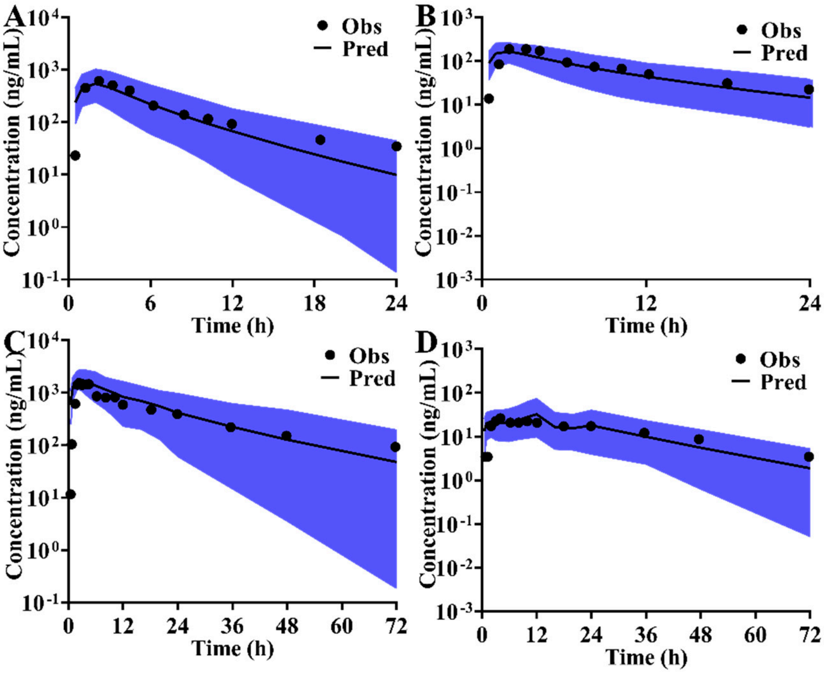

Figure S12. The validation results for ticagrelor and its activity metabolite systemic exposure in the absence ((A,B) respectively) and presence of CYP 3A strong inhibitor ketoconazole ((C,D) respectively).

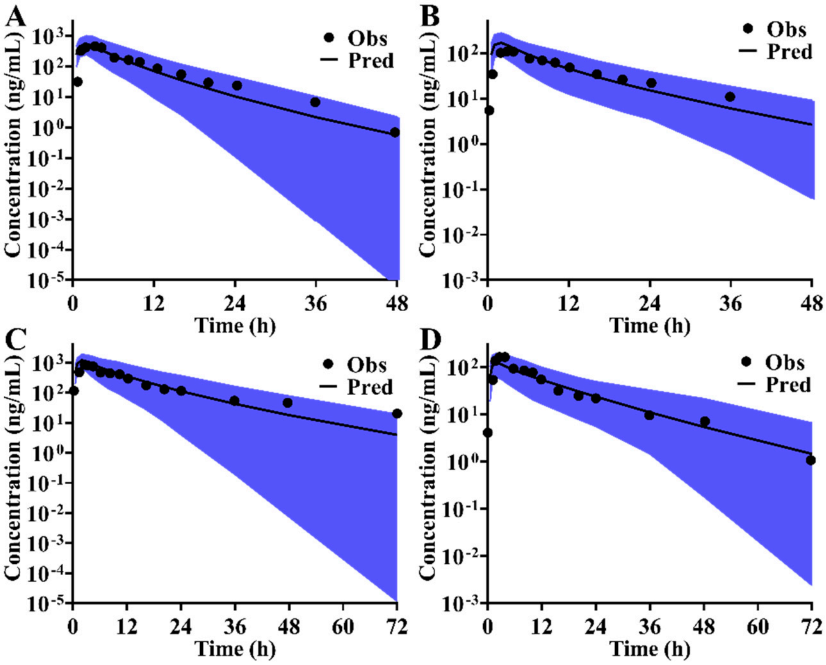

Figure S13. The validation results for ticagrelor and its activity metabolite systemic exposure in the absence ((A,B) respectively) and presence of CYP 3A moderate inhibitor diltiazem ((C,D) respectively).

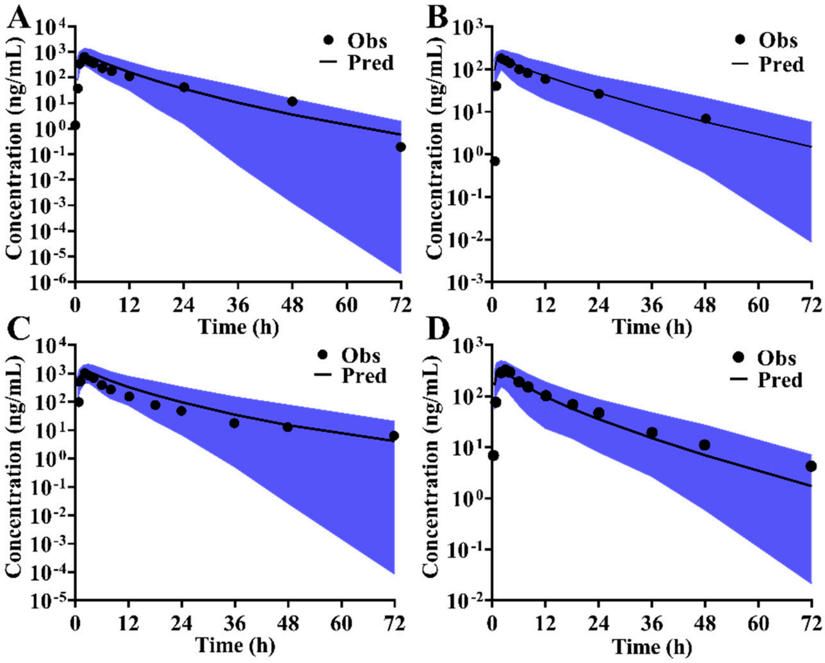

Figure S14. The validation results for ticagrelor and its activity metabolite systemic exposure in the mild hepatic impairment population ((A,B) respectively) and severe renal impairment population ((C,D) respectively).

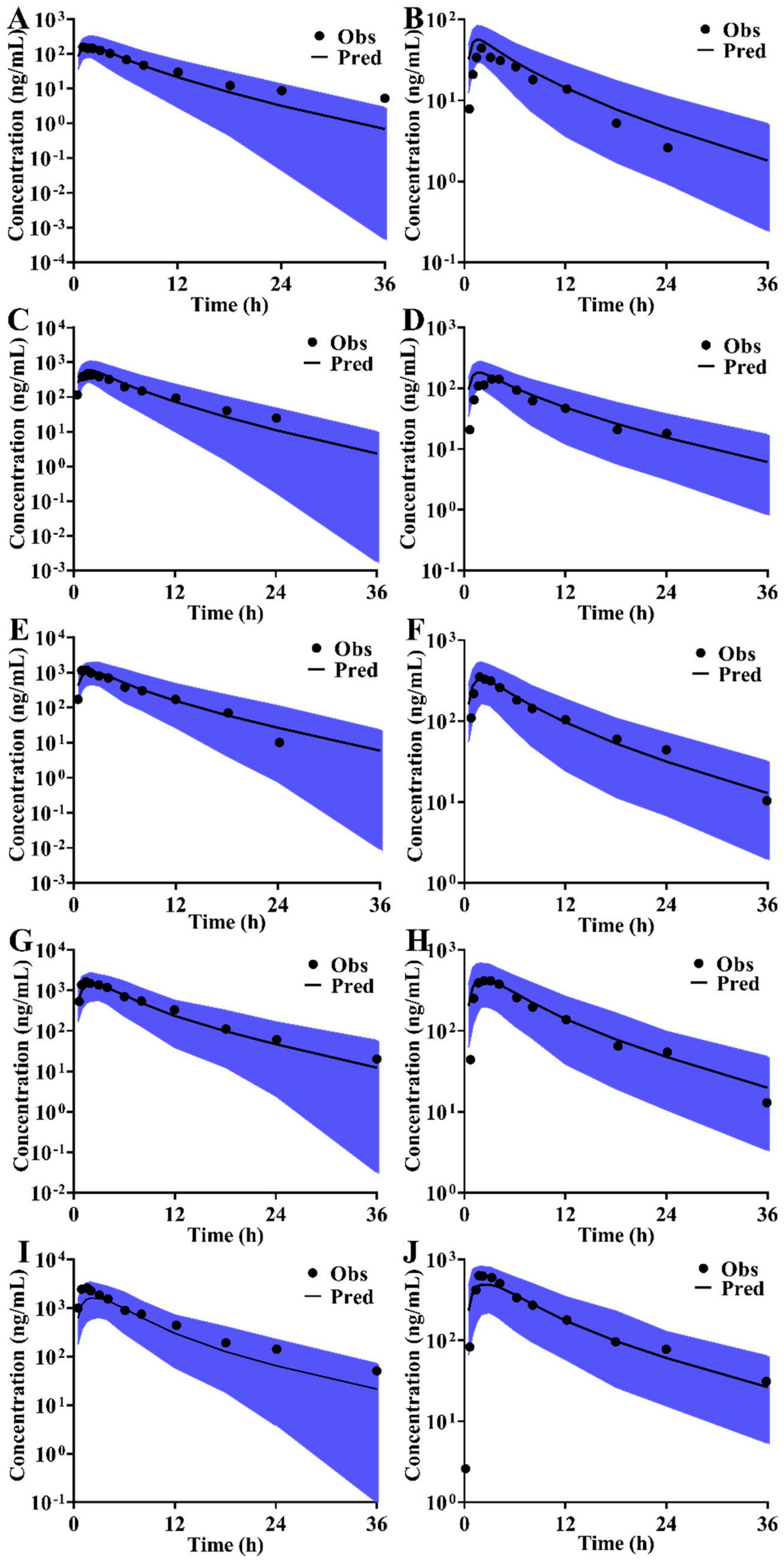

Figure S15. The validation results for ticagrelor and its activity metabolite SAD study in Caucasians ((A,C,E,G,I) representing the validation results of ticagrelor at doses of 30, 100, 200, 300 and 400 mg, respectively; (B,D,F,H,J) representing the validation results of ticagrelor activity metabolite at doses of 30, 100, 200, 300 and 400 mg, respectively).

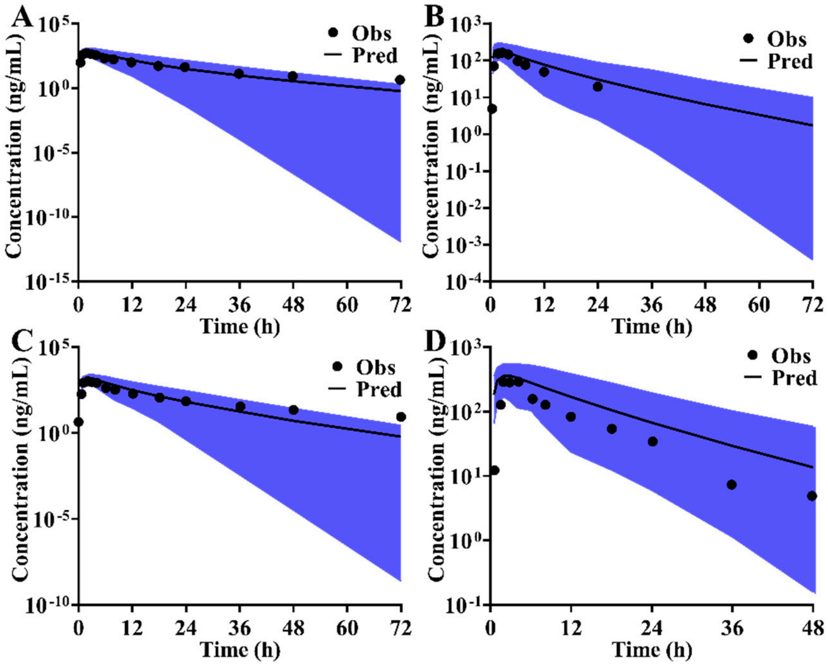

Figure S16. The validation results for ticagrelor and its activity metabolite PK study in Chinese ((A,C) representing the validation results of ticagrelor at doses of 90 and 180 mg, respectively; (B,D) representing the validation results of ticagrelor activity metabolite at doses of 90 and 180 mg, respectively).

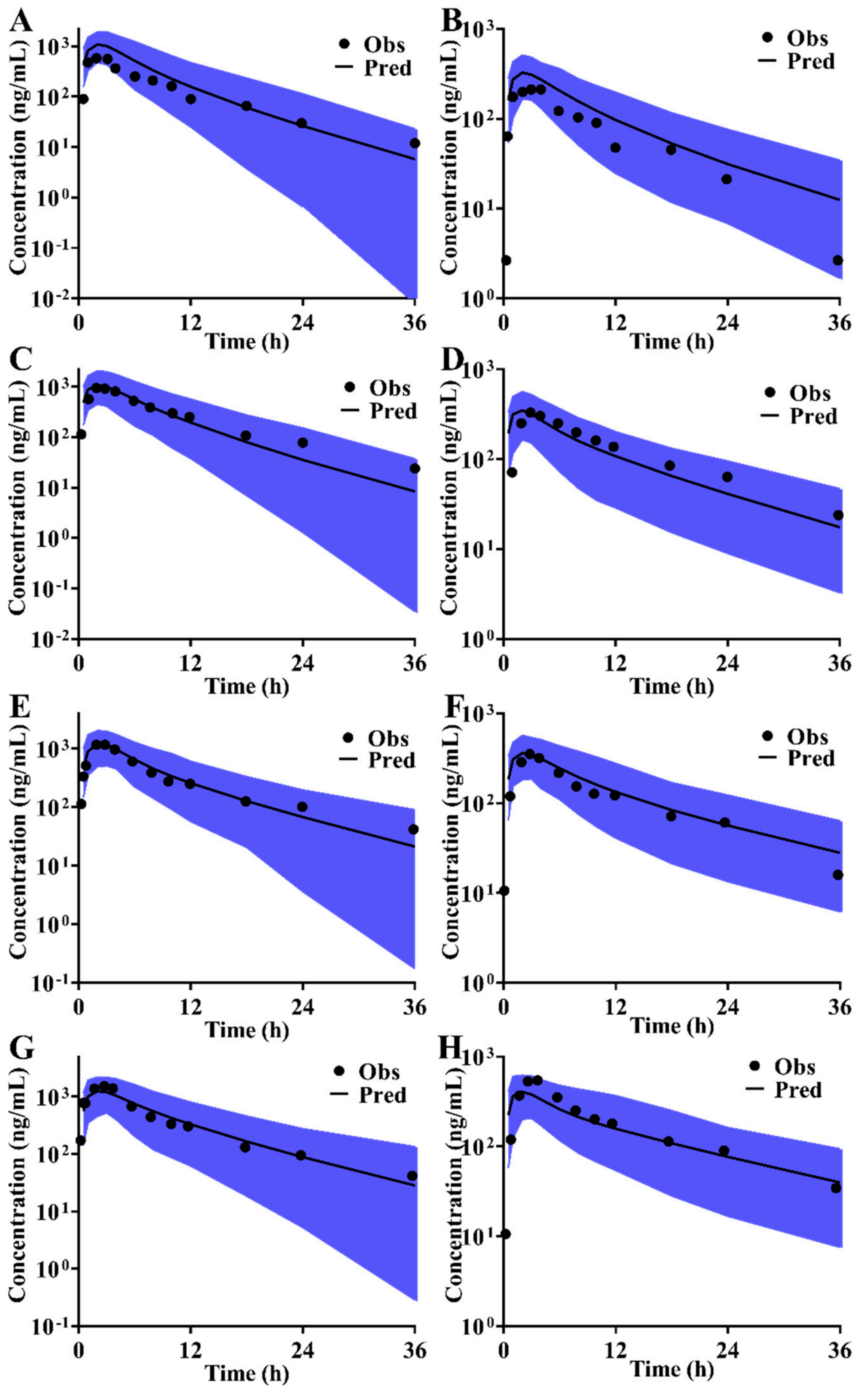

Figure S17. Validation results of ticagrelor and its activity metabolite systemic exposure in adult males ((A,B) respectively), adult females ((C,D) respectively), elderly males ((E,F) respectively) and elderly females ((G,H) respectively).

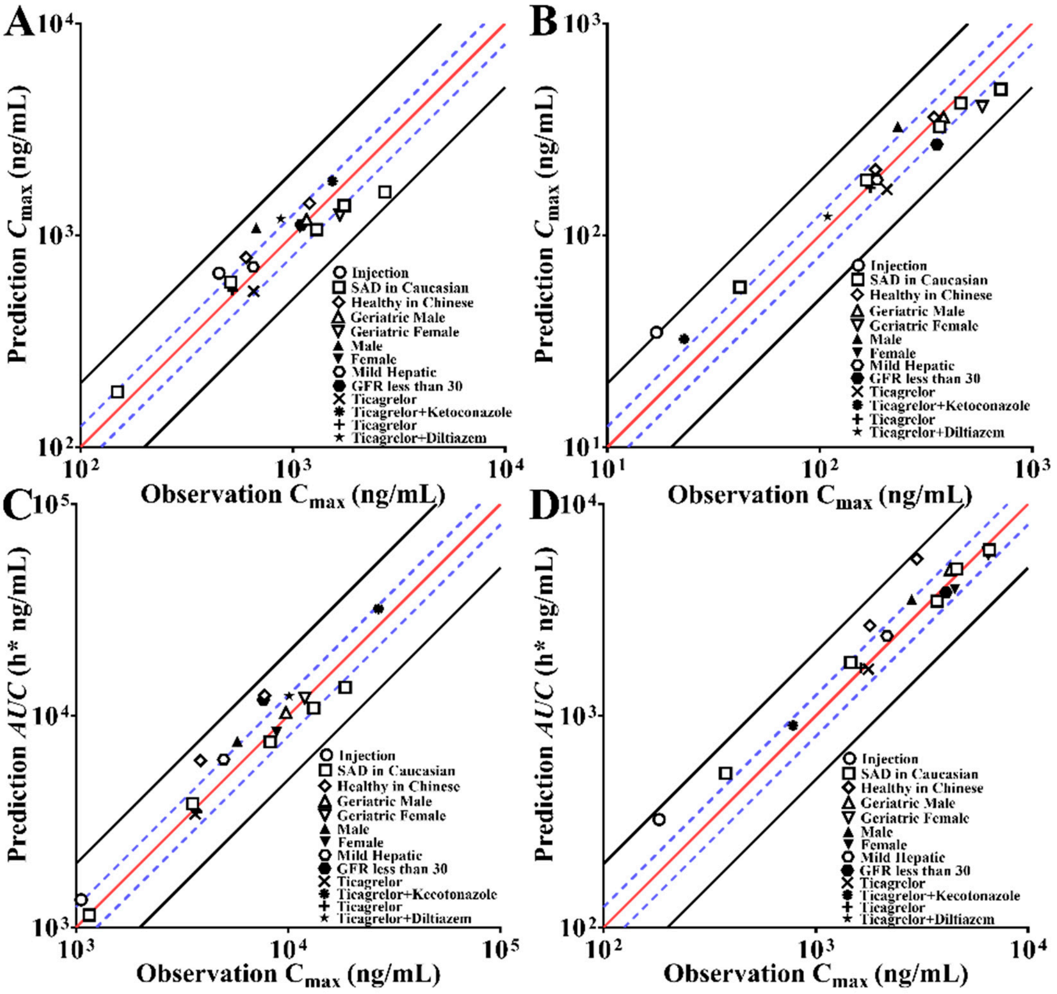

Figure S18. Pharmacokinetic parameters validated results of ticagrelor (A,C) and its activity metabolite (B,D). (The black solid lines are predefined 2-fold, the blue dashed lines are the predefined 1.25-fold boundary, and the red solid line is axle wire).

S2.2.3. Model parameters for optimized Ticagrelor PBPK model

Table S5. Parameters for optimized ticagrelor PBPK model.

Parameter	Input Value	Source
Physicochemical properties		
Molecular weight (g/mol)	522.57	
log *P*	3.521	Predicted
Compound type	Diprotic base	
pK_{a1}	2.82	Predicted
pK_{a2}	0.98	Predicted
Blood-to-plasma partition ratio	0.592	Measured [18]
Fraction unbound in plasma	0.006	Measured [18]
Absorption		
Absorption model	ADAM	
Permeability Assay	PAMPA	
PAMPA (10E-06 cm/s)	90.2 (Fasted)/ 14.30 (Fed)	Measured
DLM Particle Handling Model	Particle Population	

	Balance	
Formulation	Immediate release	
Solid State Specific Parameters	Solid state 1	
Dissolution Type	Solubility	
Solubility Type	Intrinsic solubility	
Solubility (mg/mL)	0.006	Evaluated by SIVA software
So_scalar	Global	
Global So_scalar	1.0	Defaulted
Salt Limited Solubility Model	Solubility Factors	
Solubility Factors 1	1.875	Evaluated by SIVA software
Supersaturation Precipitation Model	First order	
Precipitation Model	Model 2	
PRC	Global	
PRC (1/h)	1.00E-04	Minimum value
CSR value	1.001	Minimum value
Particle size distribution		
Radius Mean (μm)	3.957	Predicted
Minimum Radius (μm) (Distribution)	0.65	Predicted
Maximum Radius (μm) (Distribution)	13.491	Predicted
Particle density (g/mL)	1.2	Defaulted
Number of Particle Size Bins (Simulation)	30	Defaulted
Minimum Radius (μm) (Simulation)	0.100	Predicted
Maximum Radius (μm) (Simulation)	14.84	Predicted
DLM Scalar	All segments	
DLM Scalar values	0.001	
h_{eff} method selected	Hintz-Johnson	
h_{eff} cut-off value (μm)	2.00E-04	Evaluated by SIVA software
Bile Micelle mediated solubilisation	On	
Bile solubilization input type	User	
User log $K_{m:w, neutral}$	5.941	Evaluated by SIVA software
User log $K_{m:w, ion}$	8.226	Evaluated by SIVA software
Distribution		
Distribution model	Full PBPK model	
V_{ss} input type	Predicted	
Prediction Method	Method 2	
Tissue : Plasma Partition Coefficients	Predicted	
K_p scalar	1.0	Fitted
Elimination		

Clearance type	Enzyme kinetics	
Intrinsic clearance of CYP 3A4 (μL/min/pmol)	5.00	Fitted
Additional HLM CL_{int} (μL/min/mg protein)	100	Fitted
CL_R typical renal clearance (L/h)	0.00584	Measured [17]
Metabolite		
Physicochemical properties		
Molecular weight (g/mol)	478.5	
log *P*	3.58	Predicted
Compound type	Diprotic base	
pK_{a1}	2.76	Predicted
pK_{a2}	0.76	Predicted
Blood-to-plasma partition ratio	0.5706	Predicted
Fraction unbound in plasma	0.002	Measured [14]
Distribution		
Distribution model	Full PBPK model	
V_{ss} input type	Predicted	
Prediction Method	Method 2	
Tissue : Plasma Partition Coefficients	Predicted	
K_p scalar	5.5	Fitted
Elimination		
Clearance type	Enzyme kinetics	
Intrinsic clearance of CYP 3A4 (μL/min/pmol)	6.000	Fitted
Additional HLM CL_{int} (μL/min/mg protein)	5000	Fitted

S2.3. PB-201 PBPK model development and validation

S2.3.1. PB-201 PBPK model development for metabolism and elimination module

PB-201 PBPK model is an updated iteration of a previously published model [27], with the primary distinction arising from the absorption model. The original version was developed based on fitting the absorption phase of the *in vivo* C-T profiles. In this manuscript, the metabolic module of the PB-201 PBPK model remains consistent with the former [27]. The specific details are outlined below:

PB-201 undergoes elimination through both hepatic metabolism and renal excretion. The CL_{int} values for each CYP isoenzyme and CL_{HLM} were combined to characterize the *in vivo* metabolic behavior of PB-201. CL_{int} was derived from human recombinase experiments, and ISEF was incorporated into the PBPK model used to calibrate the contribution of CYP isoforms to systemic clearance. Following the verification of the contribution of CYP3A4 through a DDI study involving PB-201 and keconazole, CL_{HLM} was used to capture hepatic metabolic clearance with an uncertain metabolic mechanism. This parameter was obtained by fitting the concentration-time profiles of PB-201 in the absence and presence of keconazole. The CL_R was directly extracted from an *in vivo* clinical study and integrated into the PBPK model. The model parameters for PB-201 are provided in **Table S6**.

Table S6. Parameters for PB-201 PBPK model development.

Parameter	Input Value	Source
Physicochemical properties		
Molecular weight (g/mol)	432.44	
log *P*	2.102	Measured
Compound type	Monoprotic base	
pK_a	10.41	Measured
Blood-to-plasma partition ratio	0.77	Measured
Fraction unbound in plasma	0.303	Measured
Absorption		
Absorption model	ADAM	
Permeability Assay	Caco-2	
Apical pH : Basolateral pH	7.4 : 7.4	
Activity	Passive&Active	
Caco-2(10^{-6}cm/s) (PB-201)	8.88	Measured
Caco-2(10^{-6}cm/s) (Propranolol)	29.4	Measured
DLM Particle Handling Model	Particle Population Balance	
Formulation	Immediate release	
Solid State Specific Parameters	Solid state 1	
Dissolution Type	Solubility	
Solubility Type	Intrinsic solubility	
Solubility (mg/mL)	0.003	Evaluated by SIVA software
S_{o_scalar}	Global	
Global S_{o_scalar}	1.0	Defaulted
Salt Limited Solubility Model	Solubility Factors	
Solubility Factors 1	62.84	Evaluated by SIVA

		software
Supersaturation Precipitation Model	First order	
Precipitation Model	Model 2	
PRC	Global	
PRC (1/h)	95.966	Evaluated by SIVA software
CSR value	10000	Evaluated by SIVA software
Dispersion Type	Monodispersed	
Input Type	Volume Fraction	
Monodispersed Radius (μm)	1.697	Predicted
Particle density (g/mL)	1.2	Defaulted
Number of Particle Size Bins (Simulation)	30	Defaulted
Minimum Radius (μm) (Simulation)	0.100	Defaulted
Maximum Radius (μm) (Simulation)	11.0	Defaulted
DLM Scalar	All segments	
DLM Scalar values	0.031	Evaluated by SIVA software
h_{eff} method selected	Hintz-Johnson	
h_{eff} cut-off value (μm)	40.60	Evaluated by SIVA software
Bile Micelle mediated solubilisation	On	
Bile solubilization input type	User	
User log $K_{m:w,\ neutral}$	1.00E-06	Evaluated by SIVA software
User log $K_{m:w,\ ion}$	1.00E-06	Evaluated by SIVA software
Distribution		
Distribution model	Full PBPK model	
Predicted	Predicted	
Prediction Method	Method 2	
Tissue : Plasma Partition Coefficients		Predicted
K_p scalar	1.50	Fitted
Elimination		
Clearance type	Enzyme kinetics	
Intrinsic clearance of CYP 1A2 (μL/min/pmol)	0.068	Measured
ISEFs	0.336	
Intrinsic clearance of CYP 2B6 (μL/min/pmol)	0.072	Measured
ISEFs	1.452	
Intrinsic clearance of CYP 2C8 (μL/min/pmol)	0.06	Measured
ISEFs	1.452	
Intrinsic clearance of CYP 2C9 (μL/min/pmol)	0.144	Measured
ISEFs	0.548	

Intrinsic clearance of CYP 2C19 (μL/min/pmol)	0.113	Measured
ISEFs	1.452	
Intrinsic clearance of CYP 2D6 (μL/min/pmol)	0.145	Measured
ISEFs	0.66	
Intrinsic clearance of CYP 3A4 (μL/min/pmol)	0.141	Measured
ISEFs	0.33	Fitted
CL_R typical renal clearance (L/h)	0.099	Measured
Additional Hep CL_{int} (μL/min/10^6)	2.50	Fitted

S2.3.2. PB-201 PBPK model validation

The validation dataset of PB-201 were provided by the sponsor, including the MAD study in Caucasians and Chinese, and the DDI study about PB-201coadministration with ketoconazole. Both the observed PK profiles of PB-201 before (**Figure S19A**) and after (**Figure S19B**) its coadministration with CY3A4 inhibitor fell within the 90% CI range of the predicted profiles, and the PK ratios for AUC and C_{max} were within the 0.80- to 1.25-fold range (**Figure S22**), suggesting that the contribution of CYP3A4 to the systemic clearance of PB-201 in the PBPK model was reasonable. The predictive performance of the PB-201 was validated by data from the MAD studies, and the validated results met the two predefined criteria (**Figure S20** and **Figure S21**). Therefore, the PB-201 PBPK model can be used to explore PK properties in untested scenarios.

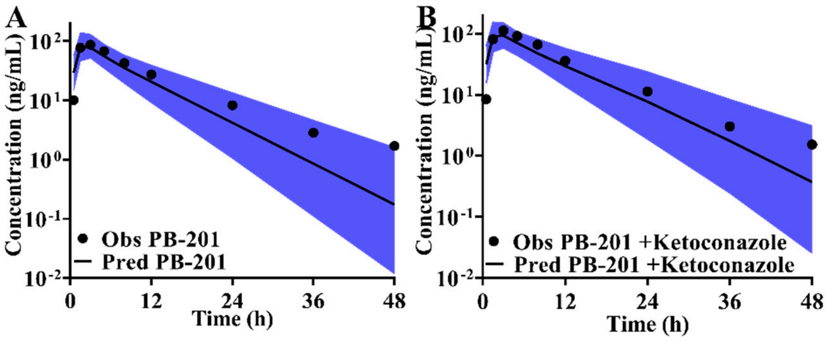

Figure S19. The validated results for PB-201 systemic exposure in the absence (A) and presence (B) of CYP 3A strong inhibitor ketoconazole.

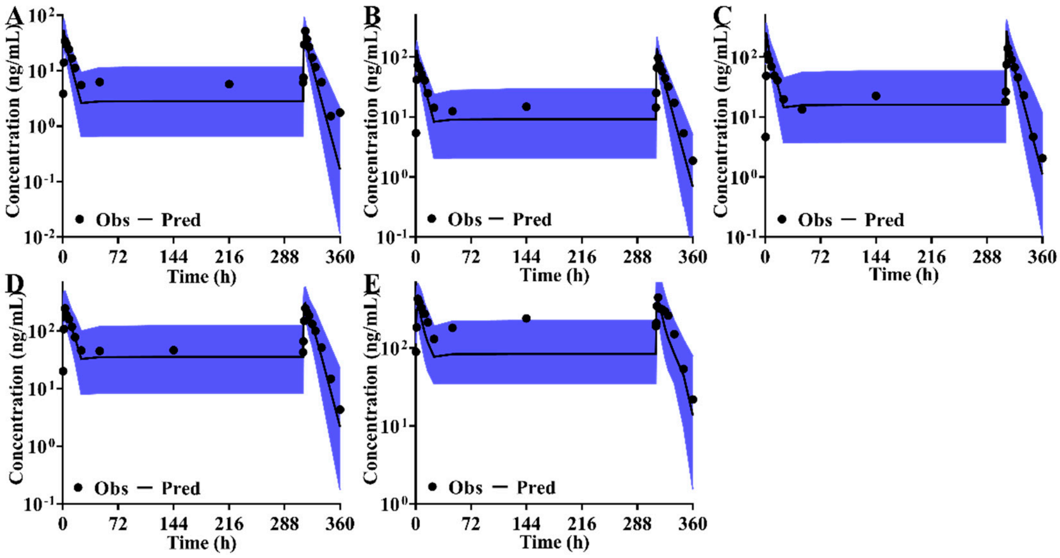

Figure S20. The validation results for PB-201 MAD study in Caucasians ((A,B,C,D,E) representing the validation results of PB-201 at doses of 10, 30, 50, 100 and 300 mg, respectively).

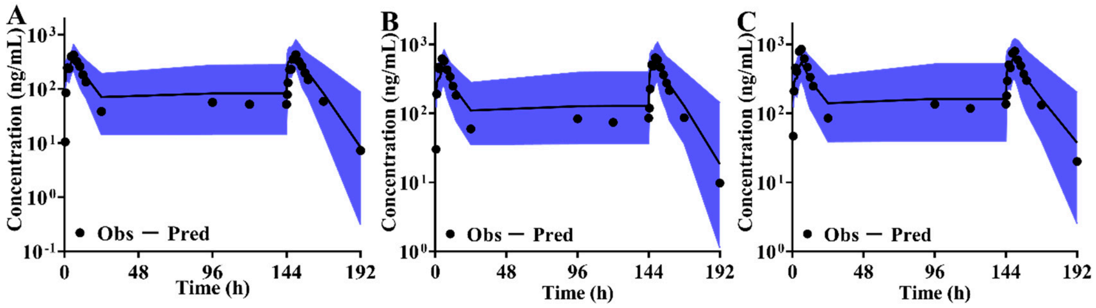

Figure S21. The validation results for PB-201 MAD study in Chinese volunteers ((A,B,C) representing the validation results of PB-201 at doses of 50 mg in the morning and 50 mg in the noon, 100 mg in the morning and 50 mg in the noon, and 100 mg in the morning and 100 mg in the noon, respectively).

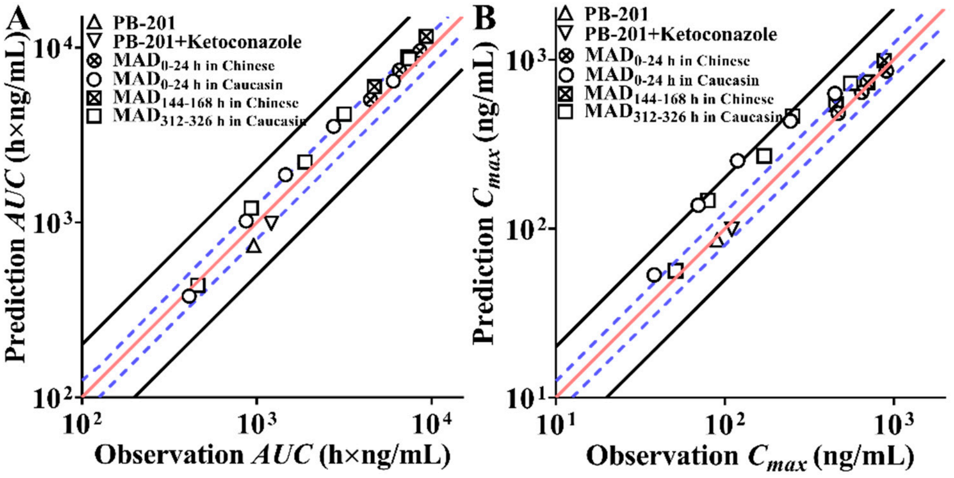

Figure S22. Pharmacokinetic parameters validated results of PB-201 (A,B). (The black solid lines are predefined 2-fold, the blue dashed lines are the predefined 1.25-fold boundary, and the red solid line is axle wire).

References

1. Weinz, C.; Schwarz, T.; Kubitza, D.; Mueck, W.; Lang, D. Metabolism and excretion of rivaroxaban, an oral, direct factor Xa inhibitor, in rats, dogs, and humans. *Drug Metab. Dispos.*. **2009**, *37*, 1056-1064, doi:10.1124/dmd.108.025569.
2. Kvasnicka, T.; Malikova, I.; Zenahlikova, Z.; Kettnerova, K.; Brzezkova, R.; Zima, T.; Ulrych, J.; Briza, J.; Netuka, I.; Kvasnicka, J. Rivaroxaban - Metabolism, Pharmacologic Properties and Drug Interactions. *Curr. Drug Metab.*. **2017**, *18*, 636-642, doi:10.2174/1389200218666170518165443.
3. Kubitza, D.; Becka, M.; Wensing, G.; Voith, B.; Zuehlsdorf, M. Safety, pharmacodynamics, and pharmacokinetics of BAY 59-7939--an oral, direct Factor Xa inhibitor--after multiple dosing in healthy male subjects. *Eur. J. Clin. Pharmacol.*. **2005**, *61*, 873-880, doi:10.1007/s00228-005-0043-5.
4. Gnoth, M.J.; Buetehorn, U.; Muenster, U.; Schwarz, T.; Sandmann, S. In vitro and in vivo P-glycoprotein transport characteristics of rivaroxaban. *J. Pharmacol. Exp. Ther.*. **2011**, *338*, 372-380, doi:10.1124/jpet.111.180240.
5. Matsuzaki, T.; Scotcher, D.; Darwich, A.S.; Galetin, A.; Rostami-Hodjegan, A. Towards Further Verification of Physiologically-Based Kidney Models: Predictability of the Effects of Urine-Flow and Urine-pH on Renal Clearance. *J. Pharmacol. Exp. Ther.*. **2019**, *368*, 157-168, doi:10.1124/jpet.118.251413.
6. De Bruyn, T.; Ufuk, A.; Cantrill, C.; Kosa, R.E.; Bi, Y.A.; Niosi, M.; Modi, S.; Rodrigues, A.D.; Tremaine, L.M.; Varma, M.; et al. Predicting Human Clearance of Organic Anion Transporting Polypeptide Substrates Using Cynomolgus Monkey: In Vitro-In Vivo Scaling of Hepatic Uptake Clearance. *Drug Metab. Dispos.*. **2018**, *46*, 989-1000, doi:10.1124/dmd.118.081315.
7. Food and Drug Administration, 2021. Rivaroxaban-Clinical Pharmacology and Biopharmaceutics Review. https://www.accessdata.fda.gov/drugsatfda_docs/nda/2022/215859Orig1s000ClinPharmR.pdf (assessed 20 December 2021).
8. Zhao, X.; Sun, P.; Zhou, Y.; Liu, Y.; Zhang, H.; Mueck, W.; Kubitza, D.; Bauer, R.J.; Zhang, H.; Cui, Y. Safety, pharmacokinetics and pharmacodynamics of single/multiple doses of the oral, direct Factor Xa inhibitor rivaroxaban in healthy Chinese subjects. *Br J Clin Pharmacol*. **2009**, *68*, 77-88, doi:10.1111/j.1365-2125.2009.03390.x.
9. Mueck, W.; Kubitza, D.; Becka, M. Co-administration of rivaroxaban with drugs that share its elimination pathways: pharmacokinetic effects in healthy subjects. *Br J Clin Pharmacol*. **2013**, *76*, 455-466, doi:10.1111/bcp.12075.
10. Kubitza, D.; Roth, A.; Becka, M.; Alatrach, A.; Halabi, A.; Hinrichsen, H.; Mueck, W. Effect of hepatic impairment on the pharmacokinetics and pharmacodynamics of a single dose of rivaroxaban, an oral, direct Factor Xa inhibitor. *Br J Clin Pharmacol*. **2013**, *76*, 89-98, doi:10.1111/bcp.12054.
11. Kubitza, D.; Becka, M.; Mueck, W.; Halabi, A.; Maatouk, H.; Klause, N.; Lufft, V.; Wand, D.D.; Philipp, T.; Bruck, H. Effects of renal impairment on the pharmacokinetics, pharmacodynamics and safety of rivaroxaban, an oral, direct Factor Xa inhibitor. *Br J Clin Pharmacol*. **2010**, *70*, 703-712, doi:10.1111/j.1365-2125.2010.03753.x.
12. Kubitza, D.; Becka, M.; Roth, A.; Mueck, W. Dose-escalation study of the pharmacokinetics and pharmacodynamics of rivaroxaban in healthy elderly subjects. *Curr Med Res Opin*. **2008**, *24*, 2757-2765, doi:10.1185/03007990802361499.
13. Jiang, J.; Hu, Y.; Zhang, J.; Yang, J.; Mueck, W.; Kubitza, D.; Bauer, R.J.; Meng, L.; Hu, P. Safety, pharmacokinetics and pharmacodynamics of single doses of rivaroxaban - an oral, direct factor Xa inhibitor - in elderly Chinese subjects. *Thromb. Haemost.*. **2010**, *103*, 234-241, doi:10.1160/TH09-03-0196.
14. Sillén, H.; Cook, M.; Davis, P. Determination of unbound ticagrelor and its active metabolite (AR-C124910XX) in human plasma by equilibrium dialysis and LC-MS/MS. *J. Chromatogr. B Analyt. Technol. Biomed. Life Sci.*. **2011**, *879*, 2315-2322, doi:10.1016/j.jchromb.2011.06.023.
15. Teng, R.; Butler, K. Effect of the CYP3A inhibitors, diltiazem and ketoconazole, on ticagrelor pharmacokinetics in healthy volunteers. *J Drug Assess*. **2013**, *2*, 30-39, doi:10.3109/21556660.2013.785413.
16. Teng, R.; Kujacic, M.; Hsia, J. Evaluation of the pharmacokinetic interaction between ticagrelor and venlafaxine, a cytochrome P-450 2D6 substrate, in healthy subjects. *Clin Ther*. **2014**, *36*, 1217-1225, doi:10.1016/j.clinthera.2014.06.024. (assessed 20 July 2011)
17. Teng, R.; Oliver, S.; Hayes, M.A.; Butler, K. Absorption, distribution, metabolism, and excretion of ticagrelor in healthy subjects. *Drug Metab. Dispos.*. **2010**, *38*, 1514-1521, doi:10.1124/dmd.110.032250.

18. Food and Drug Administration, 2011. Ticagrelor-Clinical Pharmacology and Biopharmaceutics Review. https://www.accessdata.fda.gov/drugsatfda_docs/nda/2011/022433Orig1s000ClinPharmR.pdf.
19. Marsousi, N.; Doffey-Lazeyras, F.; Rudaz, S.; Desmeules, J.A.; Daali, Y. Intestinal permeability and P-glycoprotein-mediated efflux transport of ticagrelor in Caco-2 monolayer cells. *Fundam Clin Pharmacol*. **2016**, *30*, 577-584, doi:10.1111/fcp.12219.
20. Teng, R.; Maya, J. Absolute bioavailability and regional absorption of ticagrelor in healthy volunteers. *J Drug Assess*. **2014**, *3*, 43-50, doi:10.3109/21556660.2014.946604.
21. Teng, R.; Butler, K. Pharmacokinetics, pharmacodynamics, tolerability and safety of single ascending doses of ticagrelor, a reversibly binding oral P2Y(12) receptor antagonist, in healthy subjects. *Eur. J. Clin. Pharmacol.*. **2010**, *66*, 487-496, doi:10.1007/s00228-009-0778-5.
22. Li, H.; Butler, K.; Yang, L.; Yang, Z.; Teng, R. Pharmacokinetics and tolerability of single and multiple doses of ticagrelor in healthy Chinese subjects: an open-label, sequential, two-cohort, single-centre study. *Clin Drug Investig*. **2012**, *32*, 87-97, doi:10.2165/11595930-000000000-00000.
23. Teng, R.; Mitchell, P.; Butler, K. Effect of rifampicin on the pharmacokinetics and pharmacodynamics of ticagrelor in healthy subjects. *Eur. J. Clin. Pharmacol.*. **2013**, *69*, 877-883, doi:10.1007/s00228-012-1436-x.
24. Butler, K.; Teng, R. Pharmacokinetics, pharmacodynamics, and safety of ticagrelor in volunteers with mild hepatic impairment. *J Clin Pharmacol*. **2011**, *51*, 978-987, doi:10.1177/0091270010379409.
25. Butler, K.; Teng, R. Pharmacokinetics, pharmacodynamics, and safety of ticagrelor in volunteers with severe renal impairment. *J Clin Pharmacol*. **2012**, *52*, 1388-1398, doi:10.1177/0091270011415526.
26. Teng, R.; Mitchell, P.; Butler, K. Effect of age and gender on pharmacokinetics and pharmacodynamics of a single ticagrelor dose in healthy individuals. *Eur. J. Clin. Pharmacol.*. **2012**, *68*, 1175-1182, doi:10.1007/s00228-012-1227-4.
27. Zhang, M.; Lei, Z.; Yu, Z.; Yao, X.; Li, H.; Xu, M.; Liu, D. Development of a PBPK model to quantitatively understand absorption and disposition mechanism and support future clinical trials for PB-201. *CPT Pharmacometrics Syst Pharmacol*. **2023**, *12*, 941-952, doi:10.1002/psp4.12964.
